# Changes in ^15^NO_3_^-^ Availability and Transpiration Rate Are Associated With a Rapid Diurnal Adjustment of Anion Contents as Well as ^15^N and Water Fluxes Between the Roots and Shoots

**DOI:** 10.3389/fpls.2018.01751

**Published:** 2018-12-03

**Authors:** Charline Orieux, Gilles Demarest, Marie-Laure Decau, Patrick Beauclair, Marie-Paule Bataillé, Erwan Le Deunff

**Affiliations:** ^1^Ecophysiologie Végétale Agronomie et Nutritions N.C.S., UNICAEN, INRA, EVA, Normandie Université, Caen, France; ^2^INRA FERLUS-SOERE, INRA – Auvergne Rhône-Alpes Centre, Lusignan, France; ^3^Centre Michel de Boüard et LETG-Caen Geophen, UNICAEN, CNRS, CRAHAM, LETG, Normandie Université, Caen, France; ^4^Structure Fédérative Interactions Cellules ORganismes Environnement, UNICAEN, ICORE, Normandie Université, Caen, France

**Keywords:** nitrate uptake, water uptake, transpiration, shoot capacitance, chloride, sulfate, NUE, WUE

## Abstract

**Background and Aims:** Understanding interactions between water and nitrate fluxes in response to nitrate availability and transpiration rate is crucial to select more efficient plants for the use of water and nitrate.

**Methods:** Some of these interactions were investigated in intact *Brassica napus* plants by combining a non-destructive gravimetric device with ^15^NO_3_^-^ labeling. The set-up allowed high-resolution measurement of the effects of a cross-combination of two concentrations of KNO_3_ or KCl (0.5 and 5 mM) with two different rates of transpiration controlled by the relative humidity during a day–night cycle.

**Key Results:** Results show that (1) high external nitrate concentrations increased root water uptake significantly whatever the transpiration rate, (2) nitrate translocation depended both on the rate of nitrate uptake and loading into xylem (3) dilution-concentration effect of nitrate in the xylem was mainly modulated by both external nitrate availability and transpiration rate, (4) dynamic changes in ^15^N translocation in the xylem modified shoot growth and capacitance, and (5) variations in tissue concentrations of NO_3_^-^ induced by the experimental conditions were balanced by changes in concentrations of chloride and sulfate ions. These effects were even more amplified under low transpiration condition and 0.5 mM external nitrate concentration.

**Conclusion:** Taken together, these results highlight the fine and rapid adjustment of anion contents, nitrate and water flows to changes in transpiration rate and nitrate availability during a day–night cycle. The use of this non-invasive gravimetric device is therefore a powerful tool to assess candidates genes involved in nitrogen and water use efficiency.

## Introduction

A better understanding of the dynamic interactions between nitrate and water fluxes submitted to different transpiration rates is becoming critical for agriculture because of the worldwide increase in food demand, climate change and the increase in fertilizer costs (Good et al., 2004). Unraveling these interactions requires the development of non-invasive methods avoiding excised roots and/or pressurization of the root system, in order to analyze simultaneously the dynamic interactions of water and nitrate fluxes in intact transpiring plants during a day–night cycle (Windt et al., 2006; Schulze-Till et al., 2009; Wegner, 2015). Indeed, the invasive approaches used to date on excised roots lead to the breakdown of water relations in the plant and destroy the signal flows between shoots and roots.

A literature survey shows that non-invasive methods to measure either water flow or nitrate flow already exist. So far, these methods have been used in intact plants with a low temporal resolution (day-to-week) and without the aid of ^13^NO_3_^-^ or ^15^NO_3_^-^ tracers (Shaner and Boyer, 1976; Schulze and Bloom, 1984). However, the techniques such as psychrometry, gravimetry, potometer and nuclear magnetic resonance imaging (MRI and ^1^H NMR) allow the measurements of water flow by using high temporal resolution (Van Ieperen and Madery, 1994; Kockenberger et al., 1997; Peuke et al., 2001; Li and Shao, 2003; Windt et al., 2006; Schulze-Till et al., 2009). Likewise, the use of ^13^NO_3_^-^ and ^15^NO_3_^-^ tracers and PETIS method (Positron Emitting Tracer Imaging System) allows to measure the ^15^N translocation and nitrate flow in the xylem of intact plants over short periods of time (Clarkson et al., 1996; Kawachi et al., 2002). To our knowledge, no studies have attempted to couple these methods during a diurnal cycle by increasing the temporal resolution. As a result, we do not know how both the transpiration rate and nitrate availability affect (i) the diurnal adjustment of water and ^15^N flow velocities into the xylem and (ii) modify the shoot growth and capacitance (i.e., capacity of storing and releasing water of the tissues) through adjustments in anion contents.

Although the effect of nitrate on root hydraulic conductance (*Lr*) has been described for a long time on excised roots (Radin and Boyer, 1982; Bigot and Boucaud, 1996; Hoarau et al., 1996; Clarkson et al., 2000; Gloser et al., 2007), the effect of nitrate on the water uptake and root hydraulic conductance in intact plants has recently been re-examined using the split root system in short and long-term experiments (Guo S. et al., 2002; Guo et al., 2007; Gorska et al., 2008a). Short-term experiments have shown a rapid and significant increase in water uptake and root hydraulic conductance in the portion of the roots exposed to 5 mM external nitrate concentration compared to the other portion that was perfused with 0 mM nitrate (Gorska et al., 2008a). In long-term experiments (days), the increase in root hydraulic conductance is accompanied by a significant daily increase in water uptake in nitrate fed roots (Guo S. et al., 2002; Guo et al., 2007). The nitrate effect on root hydraulic conductance has been ascribed to transcriptional and posttranscriptional aquaporins regulation, aquaporins expression being upregulated few hours (3–12 h) after nitrate treatment of nitrogen-starved plants (Wang et al., 2001; Guo et al., 2007). Others studies reported that nitrate had no effect on transcription level but increased aquaporins activity (Gorska et al., 2008a,b). In contrast, the effect of nitrate availability on transpiration rate through the stomatal conductance (*gs*) is not always coupled with *Lr* as shown by contradictory results in the literature (Radin, 1990; Guo S. et al., 2002; Guo et al., 2007; Wilkinson et al., 2007) suggesting that these processes are highly coordinated and regulated.

Similarly, nitrate concentration into the xylem has been studied in different species from exudates obtained passively after excision or invasively after root excision and pressurization (Delhon et al., 1995a,b; Carvajal et al., 1996; Herdel et al., 2001; Peuke et al., 2001; Macduff and Bakken, 2003; Górska et al., 2010). The results are often highly variable and the diurnal variations are far from being understood (Schurr, 1999; Peuke et al., 2001). These variations are attributed to different processes such as changes in the root nitrate uptake, the loading of nitrate into the xylem (Delhon et al., 1995a; Herdel et al., 2001; Peuke et al., 2001) or the dilution-concentration effect of the xylem sap caused by the daily variations in the transpiration rate and water volume flow (Emmert, 1972; Fiscus, 1977, 1986; Kawachi et al., 2002). Amongst these processes, it is likely that nitrate uptake and xylem loading can play a major role in the diurnal dynamics of nitrate translocation from root to shoot. Several studies based on ^15^NO_3_ labeling have reported that nitrate uptake (Delhon et al., 1995a; Le Deunff and Malagoli, 2014) and the expression of *At-NRT2.1* and *At-NRT1.*1 nitrate transporters genes vary diurnally (Lejay et al., 1999; Ono et al., 2000; Matt et al., 2001). Under low transpiring conditions, the daily amounts of ^15^NO_3_^-^ and the water allocated to the shoots depend on external nitrate concentrations and correlated with *Bn-NRT2.1* and *Bn-NRT1.1* genes expression (Leblanc et al., 2013; Le Ny et al., 2013). This raises the question of the location in the control points for nitrate translocation to the shoots? As assumed by some authors, nitrate loading into xylem may be a control point involved in diurnal changes in xylem nitrate concentrations (Herdel et al., 2001; Siebrecht et al., 2007). In contrast, Delhon et al. (1995b) stated that nitrate uptake is uncoupled with nitrate translocation (loading and unloading). This last assumption is supported by electrophysiological studies on barley protoplasts from xylem parenchyma cells of the root (Köhler et al., 2002). These studies have identified an anion channel, highly permeable to nitrate: *X-QUAC* (quickly activating anion conductance) that is regulated by a positive feedback during nitrate loading in the xylem.

The root hydraulic properties dependence on nitrate availability must also be reconsidered because of the nitrate effect on the total volume flow of water through xylem elements observed by ^1^H NMR method in split-root experiments (Schulze-Till et al., 2009). Indeed, NMR studies showed that nitrate treatment significantly increases the water flow rates, due to an increase in the number of xylem functional elements for water transport in the root cross section.

Taken together, these results highlight the importance of a non-destructive device to study the interactions between water and nitrate fluxes with high temporal resolution. In this study, a non-destructive gravimetric device developed by Van Ieperen and Madery (1994) was used in combination with ^15^NO_3_^-^ labeling during a day–night cycle in intact transpiring plants. It allows to analyze the water and nitrate transport interactions independently of disturbances in hydrostatic and osmotic pressure gradients induced by environmental cues such as light intensity and temperature (Rokitta et al., 1999). By keeping these parameters constant in a growth chamber, the experiments reported here raise the following questions: (i) does nitrate availability modify the root water uptake and transpiration? (ii) How do nitrate availability and the rate of transpiration affect the diurnal flow of water and nitrate to the shoot? (iii) What is the potential range of diurnal variations in nitrate concentrations in xylem sap that is experienced in response to change in the transpiration rate and nitrate availability? (iv) How do shoots adjust to large variations in ^15^N translocated by the xylem? (v) Are there compensatory responses induced by other anions when nitrate concentrations in shoot and root tissues vary greatly during the day–night cycle?

## Materials and Methods

### Plant Material and Growth Conditions

**FIGURE 1:**
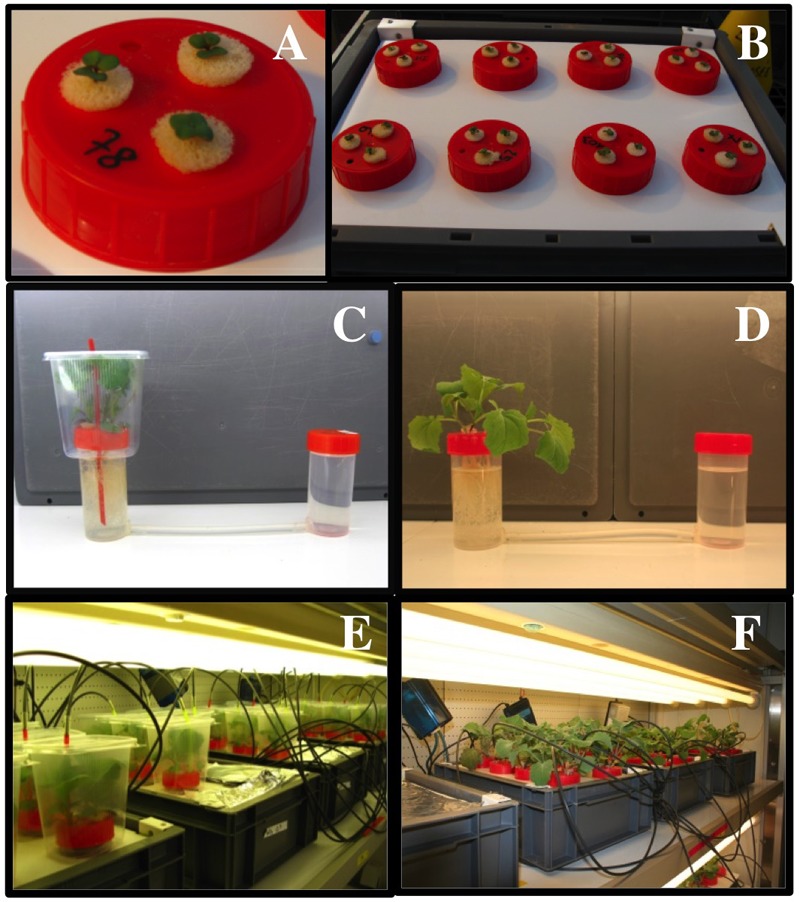
The experimental devices used to measure the rates of ^15^N uptake, transpiration and water uptake in three *Brassica napus* plants submitted to low and high transpiring conditions. **(A)** The plastic vessel unit used to measure ^15^N uptake or water uptake and transpiration on three *B. napus* plants. **(B)** Culture tank filled with 10 L of nutrient solution containing eight plastic vessel units of three plants. **(C,D)** The gravimetric device used to measure the rates of transpiration and water uptake in three *B. napus* plants submitted simultaneously to non-transpiring **(C)** and transpiring conditions **(D)**. **(E,F)** The culture system used to measure ^15^N uptake on plants fed with either 0.5 or 5 mM external nitrate concentrations and submitted simultaneously to non-transpiring **(E)** and transpiring conditions **(F)**.

**FIGURE 2:**
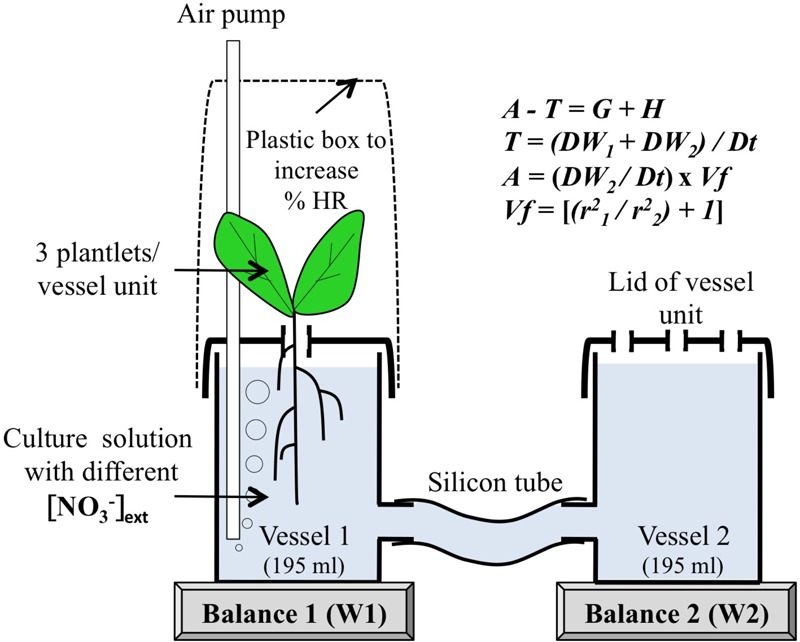
The principle of gravimetric device to measure simultaneously water uptake (*A*), transpiration (*T*) and shoot growth (*G*) and capacitance (H). *Vf* is a vessel factor depending on the radius of vessel 1 (r1) and vessel 2 (r2). Note that the bubbler constantly renews the air in the closed plastic chamber and creates a slight overpressure.

*Brassica napus* L. seeds (*cv*. Capitol) were germinated and grown hydroponically in a glasshouse and a growth chamber. The basal nutrient solution contained 0.4 μM KH_2_PO_4_, 0.15 μM K_2_HPO_4_, 1 μM K_2_SO_4_, 0.5 μM MgSO_4_, 3 μM CaCl_2_, 0.2 μM Fe-Na EDTA, 14 μM H_3_BO_3_, 5 μM MnSO_4_, 3 μM ZnSO_4_, 0.7 μM CuSO_4_, 0.7 μM (NH_4_)_6_Mo_7_O_24_ and 0.1 μM CoCl_2_, pH 6.75 and was supplemented with 1 mM KNO_3_ as the sole nitrogen source. After 6 days of germination in a glasshouse, batches of three seedlings selected according to their root length were placed upon plastic vessel units filled with 195 mL of the same nutrient solution (Figures 1A, 2). Hence, sets of twelve culture vessels open to the outside medium with holes were placed in large culture tanks filled with 20 L of basal nutrient solution with 1 mM KNO_3_ renewed every 3 days. After 25 days of culture, eight plastic vessels were transferred to small culture tanks filled with 10 L of the medium solution with 1 mM KNO_3_ (Figure 1B) and placed in a growth chamber at 22°C under a 15 h light/9 h dark photoperiod at 65% relative humidity (RH) and at a photosynthetic photon flux density of 250 μmol m^-2^ s^-1^. Air in the growth chamber was constantly renewed at a speed of 15 m s^-1^.

### Combination of Macronutrient Concentrations and Relative Humidity Treatments

Three days after their transfer into the growth chamber, all the plastic vessels previously supplied with 1 mM KNO_3_ were split into different batches to apply crossed treatments of macronutrient concentrations and relative humidity (RH; Supplementary Figure S1). Batches were acclimated for a single day–night period in a nutrient solution containing 0.5 and 5 mM KNO_3_ or KCl under ambient atmosphere (65% RH). For each macronutrient treatment: half of the batches were kept under ambient atmosphere [65% RH Figures 1D,F, high transpiration condition (HT)] whereas the others were placed under high relative humidity [100% RH Figures 1C,E, low transpiring condition (LT)]. To obtain 100% RH, for each vessel the plant shoots were enclosed in plastic chambers (Figures 1B,C, 2). The bubblers used constantly renew the air in the closed plastic chambers and create a slight overpressure (Figure 2). In order to demonstrate the performance of this system, the air temperature and relative humidity were recorded every 15 min with a HOBO data logger (U12-011, Onset Computer Corporation, Bourne, MA, United States). A comparison of the temperature and RH variations between the growth chamber and the closed plastic vessels showed that the setup allowed shoots to be maintained at 100% RH atmosphere during the course of the experiments (Supplementary Figure S2). Hence, RH values were sufficiently contrasted to compare the effects of KNO_3_ and KCl concentrations under high (HT) and low (LT) transpiration conditions in the gravimetric device (see below). Extinction of light intensity induced by plastic chambers was less than 8%. One day after applying the different combination of treatments (transpiration rates × nutrient concentrations), ^15^N (experiment 1) and water (experiment 2) fluxes were monitored in the following diel period via ^15^N labeling and fresh weight measurements, respectively (see below).

#### Experiment 1: Dynamic Measurements of ^15^N Uptake and Gain of Shoot Fresh Weight

On day 4 after transfer into the growth chamber, the two sets of culture vessels previously supplied without labeled KNO_3_ were transferred into two aerated K^15^NO_3_ solution at 0.5 or 5 mM K^15^NO_3_ for 24 h (atom % ^15^N: 5%). Net uptake of ^15^NO_3_^-^ was measured in the two sets during the day–night cycle by using series of sampling on a periodic schedule of 0, 3, 6, 9, 12, 15, 18, 21, and 24 h after treatments. All the measurements were done on six individual plants (i.e., six replicates, *N* = 6) provided by three plastic vessel units carrying 3 plants each. For each sampling time, plastic vessel units were removed from culture tanks and the shoots and roots of treated plants were separated. Then, plant organs were weighed (fresh weight) and dried (dry weight) in an oven for 72 h at 60°C. Before total N and ^15^N isotopic analyses, dry tissues were ground for 2 min to a fine powder with 5 mm diameter stainless beads in an oscillating grinder (Retsh mixer mill, MM301).

#### Experiment 2: Dynamic Measurements of Water Uptake and Transpiration

The gravimetric unit is composed of two cylindrical vessels of identical size (195 mL) connected to each other by a silicon tube (0.7 mm diameter) and filled with 330 mL hydroponic nutrient solution and aerated with an air pump according to Van Ieperen and Madery (1994) as shown in Figure 2. The gravimetric measurements were based on discontinuous weight records of the two communicating plastic vessel units using series of sampling on a periodic schedule of 0, 1h30, 3h, 4h30, 6h, 7h30, 9h, 10h30, 12h, 13h30, 15h, 16h30, 18h, 19h30, 21h, 22h30, and 24 h during one day–night cycle with a 15/9 h light-night regimen. As shown in Figure 2, for the measurements vessel unit 1 was placed on the balance 1 and vessel unit 2 without plants was placed on the balance 2 (Metler Toledo balances, XS204). For each combination of treatments and sampling time, four gravimetric units (i.e., four replicates, *N* = 4) of three plants were used per treatment. In addition, two vessel units without plants were used as controls to measure mean evaporation during the experiment. Between each measurement time, units were returned to the culture box in order to shield roots from light effects. At harvest (end of day–night cycle), shoots and roots were sampled and weighed separately, root length and leaf surface area were measured.

### Principles of Water Absorption and Transpiration Rate Measurements

The measurements were based on the equation of the water balance required for mass conservation at the whole plant level. This equation is characterized as followed (Boyer, 1985):

(1) A−T=G+H

Where *A* and *T* are the fluxes for water absorption and transpiration, *G* is the storage flux for growth and *H* is the tissue capacitance. The tissue capacitance corresponds to buffer reservoirs capable of storing and releasing water (Aroca et al., 2011; Sellin et al., 2011). According to Van Ieperen and Madery (1994), in the gravimetric device *T* and *A* values are calculated by the following equations:

(2) $${T = (DW}_{1}{+ DW}_{2})/Dt(ing{.min}^{-}{}^{1})$$

(3) $$A=(DW_{2}\times Vf)/Dt(ing.min^{-}{}^{1})$$

(4) $$Vf=[(r^{2}{}_{1}/r^{2}{}_{2})+1]$$

Where *W*_1_ = weight on balance 1 (vessel with plants), *W*_2_ = weight on balance 2 (vessel without plants), *Dt* = time interval between two measurements (min), *Vf* = vessel factor (without units) and *r*_1_ and *r*_2_ = vessel radius (cm). Because in our case the two cylindrical vessels were of an identical dimension *Vf* was equal to 2. In this set-up, the daily increase in shoot fresh weight could be caused either by the shoot growth and/or by the variation in shoot capacitance. Indeed, as previously discussed by Van Ieperen and Madery (1994), this method cannot separate growth (*G*) and tissue capacitance (*H*) from the equation 1.

### Exploratory Root Length and Leaf Surface Area Analyses

To quantify the combined effects of nutrient (KNO_3_ and KCl) concentrations and transpiration (changes in RH), modifications of the exploratory root system (primary and lateral roots) and shoot area were measured at *T*_0__h_ and *T*_24__h_ for experiment 1 and 2. The root length was analyzed by the WinRHIZO scan system (Regent Instruments Inc., Canada) and the shoot surface area with a Li-COR 3000 area meter (Inc., Lincoln, NE, United States).

### Net K^15^NO_3_^-^ Uptake and Isotope Analysis

The net uptake of NO_3_^-^ was obtained by continuous and homogeneous labeling with K^15^NO_3_ (atom % ^15^N: 5%) over 24 h. At each time point (0, 3, 6, 9, 12, 15, 18, 21, and 24 h) plants were harvested and ^15^N accumulated within the tissues analyzed by elemental analyser (analyser (EA 3000, Eurovector, Milan, Italy) coupled with an isotope ratio mass spectrometer (Isoprime X, GV instrument).

### Estimates of Changes in Xylem Nitrate Concentrations and Nitrate Supplied by Mass Flow

Estimates of the amounts of nitrate supplied to the root by apparent mass flow during a day–night cycle is calculated from the value of water uptake per cm of root length between two time points of the diurnal kinetic and nitrate concentration of the external solution (in mL 3h^-1^ cm^-1^ root) according to Bath et al. (1979):

(5) $$NMF_{ti}=[WU_{ti}/(totalrootslength\times Dt_{i})]\times {\left[NO_{3}{}^{-}\right]}_{\mathrm{ext}}$$

Where *NMF_ti_* is the average nitrate supplied by mass flow between *t_i-1_* to *t*_i_. *WU_ti_* represents water uptake between *t_i-1_* to *t*_i_ and *Dt_i_* is the time interval between two-measure points *t*_i-1_ to *t*_i_ (3 h).

Diurnal variations in nitrate concentration of xylem sap were estimated from ratio of first derivatives between of total amounts of ^15^N translocated to the shoots (in μmoles ^15^N h*^-^*^1^ plant*^-^*^1^) and the water uptake rate (in mL water h*^-^*^1^ plant*^-^*^1^) during the same time intervals of the kinetic according to following equation:

(6) $${\left[Xyl\right]}_{ti}\;=\;[{(}^{15}N_{ti}{{}_{-}}_{1}\;-{\;}^{15}N_{ti})]/[(Dt_{i}.15)/(DW_{2}\;\times \;Vf)/(3\;\times \;1000\;\times \;Dt_{i})]$$

Where Xyl*_ti_* is the xylem concentration (in mM L^-1^) between *t_i_*_-_*_1_* to *t_i_*.*_._*^15^N*_ti_* is the average of ^15^N accumulated in shoots (in μg) between *t_i_*_-_*_1_* to *t_i_*. The term (*DW_2_* ×*Vf)/3* represents the total volume of water uptake by one single plant (in μL) measured from vessel unit 2 (see Eq. 3). The factor 15 is the molecular mass of nitrogen to convert units from μg to μmole, the factor 1000 allows to convert units from μL to mL and factor 3 is used to reduce water uptake from 3 plants to one plant. The estimates of xylem sap concentrations of nitrate were made by assuming that 100% of the ^15^NO_3_ absorbed by the roots was exported under nitrate form to the shoot. However, it should be noted that previous experiment in *Hordeum vulgare* and *Zea mays* have demonstrated that only 73 and 80% of ^15^NO_3_- absorbed by the roots were assimilated in the shoots (Gojon et al., 1986). Here, the goal is not to determine the exact nitrate concentrations into the xylem but to establish by using a high temporal resolution, the diurnal dynamic of water and ^15^N translocation.

### Anion Analyses and Determination of the ^15^N/^14^N Isotopic Ratio of Nitrate

Anions such as nitrate, sulfate, chloride, phosphate and malate were extracted from fresh root and shoot tissues of three plants (*N* = 3), tissues were first frozen in liquid nitrogen then dried by lyophilisation (GT2 Basic Type 7, SRK System Technik, Gmbh). Extraction of freeze-dried tissues was carried out twice on 20 mg of the dry weight powder suspended in 1 mL of 80% ethanol then extracted at 80°C for 15 min. After centrifugation (20,000 *g* for 10 min), the supernatant was removed and the pellet was re-extracted twice in 1 mL of milliQ water for 15 min at 60°C. After centrifugation, all the supernatants were pooled and dried under vacuum then suspended into 990 μL of milliQ water. A 200 μL aliquot was taken for analyses by ion chromatography using an ICS3000 analyser (Dionex, Jouy en Josas, France) with a hydroxide-selective anion exchange column (IonPac AS17). Sample concentration was optimized in 1 mL aliquots prior to electrolytic production of high purity potassium hydroxide eluent from water with an EG40 Eluent Generator by using a range of concentrations from 12 to 40 mM. In comparison to non-suppressed ion chromatography, electrolytic and chemical suppression (ASRS300 4 mm) greatly enhanced sensitivity (signal-to-noise ratio) by decreasing the background conductivity of the eluent while simultaneously increasing the response of the analytes. In the remaining 790 μL sample, organic acids and nitrate were separated from soluble proteins, amino acids and NH_4_^+^ on a 5mL Dowex 50 H^+^ column. The column was rinsed five times with 1ml of milliQ water. After the separation, the filtrate was evaporated to dryness under vacuum at 30°C before being suspended in 200 μL of milliQ water. This fraction was then deposited on Chromosorb, packed in tin cups and dried in an oven at 55°C before the determination of the ^15^N/^14^N isotopic ratio by IRMS analysis.

### Statistical Analysis

Statistical analyses were done with the Windows version 17.3.2 of Minitab statistical software. Comparisons of means were performed with the non-parametric test of Kruskal–Wallis. Then, the Mood median test was used to compare means or medians; bars sharing different letters are significantly different at *P* < 0.05 (Sokal and Rohlf, 1995). Some measured variables were also subjected to one-way ANOVA to test significant difference between KNO_3_ and KCl treatments and/or transpiration conditions. Before ANOVA, normality and homogeneity of data were, respectively, tested with the Kolmogorov-Smirnov and Levene tests. Comparison of means was performed with the test of Tukey Honest Significant Differences at a confidence level of 0.95. The number of independent replicates is indicated in each table and figure legend. The regression coefficients were given by Excel 2008 version 12.0. The significance for each regression coefficient was obtained for DF = N-2 in Table A11 of Snedecor and Cochran (1957).

## Results

### Evaluation of the Gravimetric Device for Simultaneous Measurements of the Root Water Uptake Rate, Shoot Transpiration Rate and Gain of Shoot Fresh Weight

**FIGURE 3:**
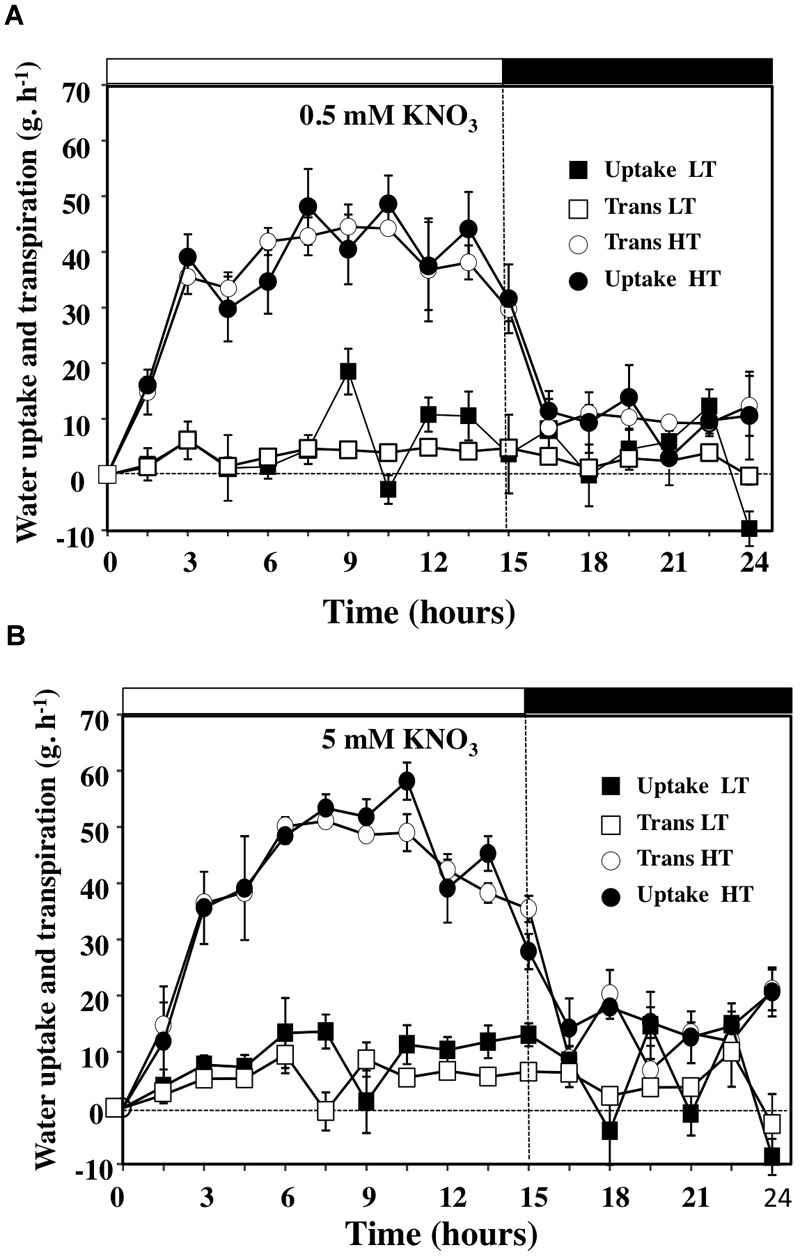
Diurnal courses of water uptake and the transpiration rates of *B. napus* plants subjected to different combinations of high and low transpiration rates (HT and LT) and 0.5 **(A)** or 5 mM **(B)** external nitrate concentrations. Values are the average (±SE) of four repeats (*N* = 4) of three plants each. Open and filled boxes indicate the light and dark periods, respectively.

The measurements performed with the gravimetric method were based on discontinuous weight records. Therefore with a single gravimetric device, it was possible to test a cross combination of KNO_3_ and KCl concentrations and two levels of transpiration rate and to obtain replicates for the measurements. Outputs given by the recordings are presented in Figure 3 for the 0.5 and 5 mM KNO_3_ treatments in high (HT) and low (LT) transpiring conditions and in Supplementary Figure S3 for the KCl treatments. Comparison of the diurnal patterns showed that water uptake and transpiration rates were parallel over the day–night period (Figures 3A,B and Supplementary Figure S3). The daily patterns obtained in HT conditions were in agreement with the continuous weight records data of Van Ieperen and Madery (1994). The strong reduction in water and transpiration rates observed in LT conditions (Figures 3A,B) demonstrates that the use of this gravimetric device is relevant for accurate analysis of relationships between the flows of water and ^15^NO_3_ for shoot growth in relation to the driving forces when temperature and light intensity are stable. Likewise, parallel measurements of leaves transpiration rate with the LiCor 6400 system in HT conditions during the light period are in accordance with the transpiration values obtained on the overall shoot with the gravimetric system (Supplementary Figure S4).

### Changes in Transpiration Rates Revealed Significant Differences Between KNO_3_ Treatments in the Daily Water Flows

**FIGURE 4:**
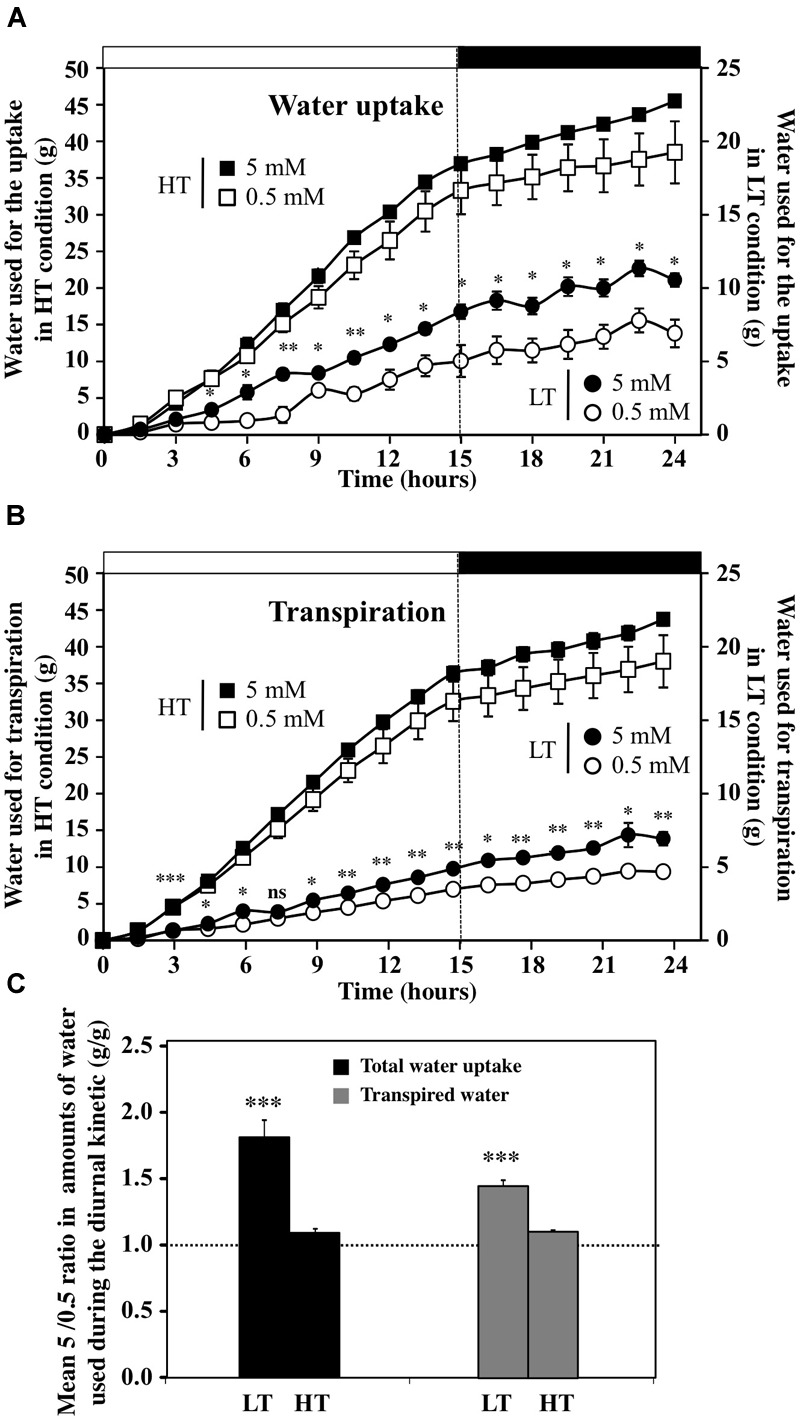
Diurnal accumulation in the amounts of water used for the uptake **(A)** and transpiration **(B)** of *B. napus* plants subjected to different combinations of transpiration rates (high and low) and fed with 5 and 0.5 mM external nitrate concentrations. Values are the average (±SE) of four repeats (*N* = 4) of three plants each. Significant differences between water uptake and transpiration under LT and HT treatments are reported; *t*-test ^∗^*P* < 0.06, ^∗∗^*P* < 0.01, ^∗∗∗^*P* < 0.005. Open and filled boxes indicate the light and dark periods, respectively. **(C)** Comparison of the mean ratios between values obtained at 5 and 0.5 mM KNO_3_ for water uptake and transpiration in plant treated with high and low transpiration rates. The mean ratios are established for each time point of the diurnal course (*N* = 15, 1.5 h time interval). Significant differences are reported for comparison between treatments; *t*-test ^∗∗∗^*P* < 0.00005.

Although the overall trends in water uptake and transpiration rates in response to an increase in external nitrate concentration were similar over the day–night cycle for all the treatments (Figure 3 and Supplementary Figure S3), cumulative water uptake was significantly higher than the amount of transpired water only in LT conditions (Figures 4A,B). Thus, the LT conditions allowed to externalize the nitrate effects on water relationships within plants normally hidden by the high transpiration stream under HT conditions (Figures 4A,B). Indeed, in HT conditions, close relationships were observed between the accumulation of water by uptake and the losses by transpiration, independent from nitrate treatments (Figures 4A,B). The differences between uptake and transpiration were only due to water accumulation in the shoot for growth and/or capacitance (see below Figure 6). However, there was about a one order of magnitude greater volume of water transpired in HT compared to LT conditions (compared primary and secondary *y*-axes, Figures 4A,B). In LT conditions, results have revealed that a residual transpiration was present at 100% RH probably caused either by the leaves guttation or air leakages induced by the slight overpressure in the closed plastic chambers (Figures 2, 4B). To better determine the effect of nitrate availability on water uptake and transpiration, we analyzed the average ratio between the 5 and 0.5 mM nitrate treatments under HT and LT conditions (Figure 4C). The average ratio was calculated from individual ratio at each time points throughout day–night cycle (Figure 4C and Supplementary Table S1). When the mean of the ratios was close or equal to 1 we considered that nitrate availability did not interact with water uptake and transpiration whereas in other cases nitrate exerted a positive (ratio >1) or negative control (ratio <1). Comparison of KNO_3_ treatments showed that the 5 mM KNO_3_ treatment induced significantly higher water uptake and residual transpiration than 0.5 mM KNO_3_ treatment under LT condition (Figure 4C).

### Gain in Shoot Fresh Weight Is Mainly Due to Changes in Shoot Capacitance

**FIGURE 5:**
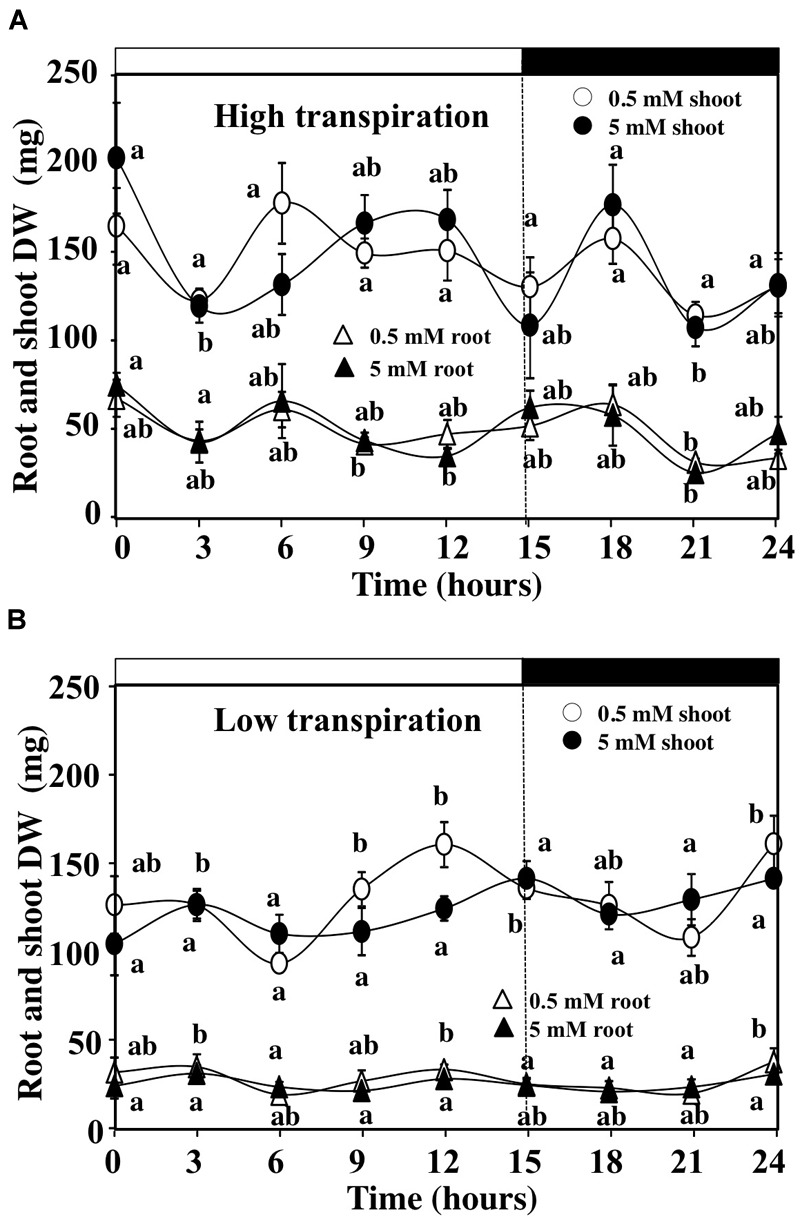
Daily changes in the shoots and roots dry weight estimated from destructive experiment. **(A)** Evolution of the shoots and roots dry weight of *B. napus* plants fed with 5 and 0.5 mM external nitrate concentrations at high transpiration rate. **(B)** Evolution of the shoots and roots dry weight of *B. napus* plants fed with 5 and 0.5 mM external nitrate concentrations at low transpiration rate. Values are the average (±SE) of six individual plants (*N* = 6).

Because the gravimetric device does not separate growth (G) from tissues capacitance (H) in equation 1, we measured the changes in the root and shoot dry weight during the time course of experiment in HT and LT conditions (Figure 5). Lack of significant difference in dry weight between the beginning and the end of the day–night cycle for the different conditions used (Figures 5A,B) demonstrates that the gain in shoot fresh weight was probably due to changes in the shoot capacitance rather than shoot growth in response to nitrate availability. The capacitance is defined as the water storage capacity of the tissues that influences the general metabolism of the plant. In shoots, water can be stored in the xylem and the leaf parenchyma. The regulation of shoot capacitance allows the modulation of hydraulic relations by buffering changes in the xylem pressure induced by high and low transpiration (Meinzer and McCulloh, 2013; Zeppel et al., 2014). Therefore, we calculated differences in the shoot growth and capacitance between the treatments to study the combined effects of external nitrate concentrations and transpiration rate on the shoot responses.

### Low Transpiring Conditions and High Nitrate Concentrations Lead to a Significant Gain in the Shoot Capacitance

**FIGURE 6:**
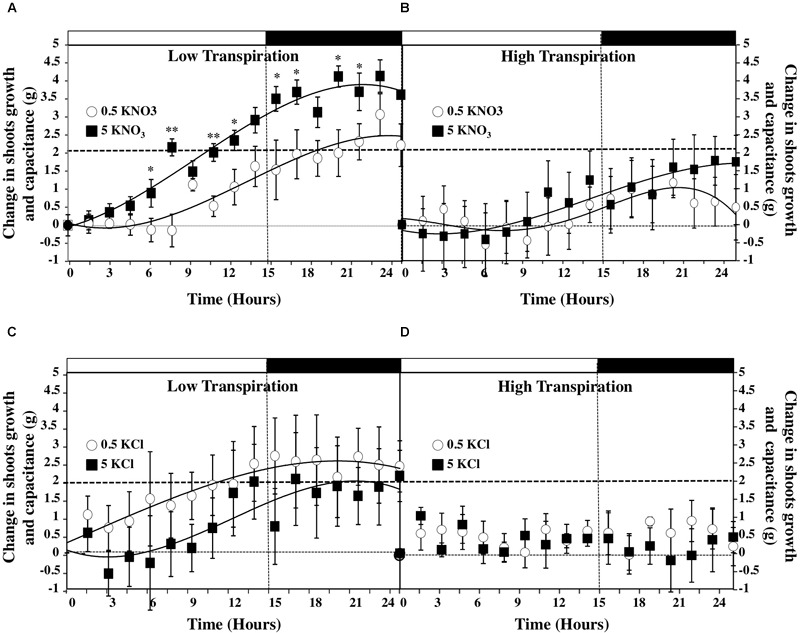
Diurnal changes in the shoots growth and capacitance of *B. napus* plants subjected to different combinations of transpiration rates low **(A,B)** and high **(C,D)** and external KNO_3_ **(A,D)** and KCL **(C,D)** concentrations (0.5 and 5 mM). Values are the average (±SE) of four repeats (*N* = 4) of three plants each. Curves were fitted by polynomial function according to the method of least squares. Open and filled boxes indicate the light and dark periods, respectively. Significant differences are reported for comparison between LT and HT treatments; *t*-test ^∗^*P* < 0.06, ^∗∗^*P* < 0.01.

According to the equation (1), the volumetric changes of the osmotic flow of water for growth and capacitance were determined by integration of the water gain per hour over time (Figure 6). Estimates showed that compared to LT conditions (Figures 6A,C), HT conditions greatly reduced shoot growth and capacitance in plants treated with high and low salt concentrations (Figures 6B,D). By contrast, under LT conditions gain in shoot fresh weight was the highest at the end of the night period after plants treatment with 5 mM KNO_3_ (Figure 6A). Moreover, the comparison of KNO_3_ and KCl treatments in LT (Figures 6A,C) and HT (Figures 6B,D) conditions indicated that changes in shoot growth and capacitance observed between KNO_3_ treatments was mainly caused by nitrate instead of potassium. Furthermore, we calculated the changes in water accumulation rate by derivation of the fitting curves of water accumulation to compare the changes in water accumulation rate for growth and capacitance during the day–night cycle (Figure 6 and Supplementary Figure S5). The profiles of water accumulation rates revealed that the lag period to reach the maximum rate of water accumulation was shortened by 5 mM nitrate treatment whilst the amplitude and the duration of the water uptake rate were significantly increased in LT conditions (Supplementary Figure S5).

### Values of Water Uptake and Transpiration Based Upon Total Root Length and Shoot Surface Area Confirm Nitrate Effect on the Water Uptake and Translocation

**FIGURE 7:**
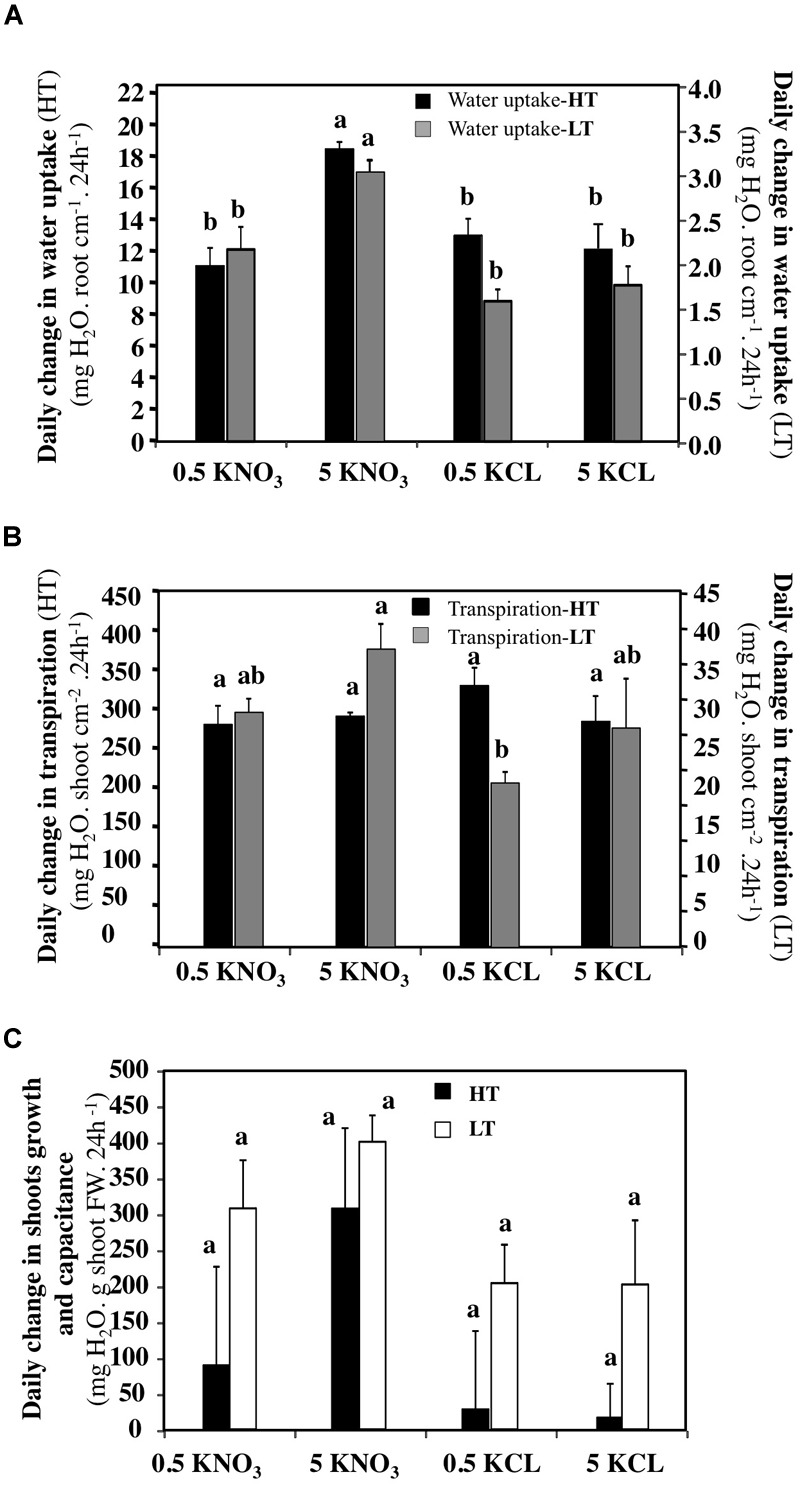
Daily changes in normalized water uptake, transpiration and shoots growth and capacitance of *B. napus* plants subjected to different combinations of transpiration rates (high and low) and external KNO_3_ and KCL concentrations (0.5 and 5 mM). **(A)** Daily changes in water uptake expressed in centimeter of root length. **(B)** Daily changes in the shoots transpiration expressed in cm^2^ of shoot surface area. **(C)** Daily changes in the shoots capacitance expressed as g of shoot fresh weight. Values are the average (±SE) of four repeats (*N* = 4) of three plants each. Different letters above error bars designate statistically significant differences between means of the different treatments (Tukey HSD test, *p* ≤ 0.05).

To compare the effects of the treatments more accurately, the daily amount of water uptake, transpiration and gain in shoot biomass were expressed by shoot surface area (cm^2^), total root length (cm) and shoot biomass (g) of the treated plants (Figure 7). Analyse of variance confirmed that whatever the transpiration rate, a 5 mM KNO_3_ treatment increased water uptake significantly (Figure 7A). In contrast, the data showed that LT conditions induced a significant increase of transpiration in response to 5 mM KNO_3_ treatment (Figure 7B). However, the increase in shoot capacitance induced by 5 mM KNO_3_ was greater in LT (Figure 7C). Because the effects of the KCl treatments at 0.5 and 5 mM were comparable to the 0.5 mM KNO_3_ treatment, the KCl treatment data are not presented in the following results.

### A High Transpiration Rate Amplifies the Positive Effect of a High External Nitrate Concentration on K^15^NO_3_ Absorption and ^15^N Translocation

**FIGURE 8:**
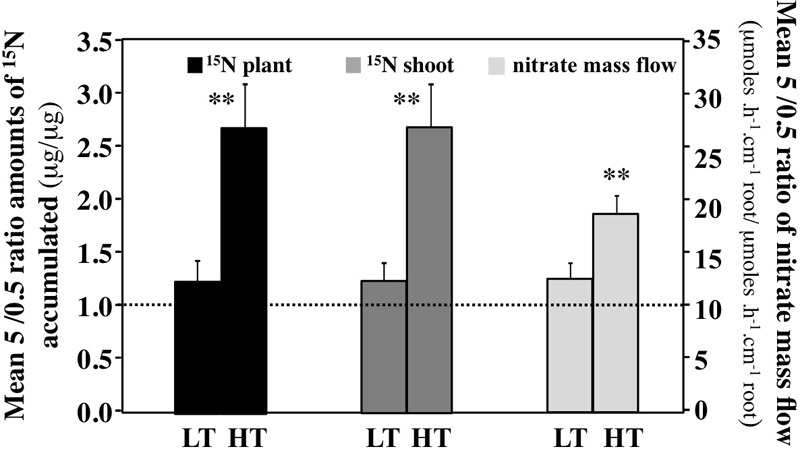
Comparison of the mean ratios between values obtained at 5 and 0.5 mM K^15^NO_3_ in plants treated with high and low transpiration rates. The ratio for total ^15^NO_3_^-^ uptake and ^15^N translocation to the shoot are calculated from each time point of the diurnal kinetic (3 h time interval) and the average is calculated for *N* = 8 values. For the nitrate mass flow, the mean ratios for each time point of the diurnal kinetic are calculated for 1.5 h time interval and the average is calculated for *N* = 15 values. Values used are the average (±SE) of four repeats (*N* = 4) of three plants each. Significant differences are reported for comparison between LT and HT treatments; *t*-test ^∗^*P* < 0.01, ^∗∗^*P* < 0.005.

To further determine the effect of transpiration rates on K^15^NO_3_ absorption and ^15^N translocation in relation to nitrate availability, the average ratio of ^15^N amounts accumulated between the 5 and 0.5 mM nitrate treatments under HT and LT conditions were calculated at each time points of the time series (Figure 8 and Supplementary Tables S2A,B). These mean ratios account for the differences in net absorption rates of nitrate caused by the differential functioning of nitrate transport systems HATS (High Affinity Transport System) and LATS (Low Affinity Transport System) during the kinetics. It is expected that at 5 mM nitrate, both transport systems (HATS + LATS) can operate at the same time, while at 0.5 mM only the HATS transport system can operate. Therefore, the mean ratio 5/0.5 account for the differential net uptake rate activity of the transport systems according to the equation: (HATS + LATS)/HATS possibly modified by the transpiration rate. The ratio between 0.5 and 5 mM influx rate values estimated from the nitrate isotherms of *B. napus* and *Arabidopsis* gives us an order of magnitude of the expected value (Filleur et al., 2001; Faure-Rabasse et al., 2002). This value is between 1.7 <average ratio 5/0.5 <2.5. The results revealed that LT conditions induced a significant decrease in ^15^N net uptake rate between 5 and 0.5 mM treatment compared to HT conditions (Figure 8). This suggests that at 5 mM under LT conditions either the LATS activity is down regulated or/and the HATS activity is up regulated since at 0.5 mM, only the HATS is able to operate. As expected, a closer examination of the ^15^N amounts accumulated showed that in 0.5 mM nitrate treatment the HATS activity increased more significantly under LT conditions than under HT conditions (compare 0.5 nitrate treatment under HT and LT conditions, Supplementary Tables S2A,B). The LT conditions at 5 mM nitrate provoked a collapse in the LATS activity (compare 0.5 and 5 mM treatment under LT conditions in Supplementary Table S2B). However, the estimates of ^15^N translocation to the shoots showed no significant difference between HT and LT conditions (Supplementary Figure S6). Both results indicate that the major part of ^15^NO_3_^-^ taken up by the roots is translocated to the shoots and that ^15^NO_3_^-^ taken up is positively correlated with the high volumes of water absorbed and translocated. This assumption is also confirmed by the estimates of nitrate supplied to the roots by apparent mass flow that follow a diurnal regulation modulated by the volume of water taken up and translocated (Supplementary Figures S7A,B). Conversely, a reduction in the rate of transpiration influences negatively the rate of net nitrate uptake as a function of nitrate availability.

### Estimates of Diurnal Changes in Xylem Nitrate Concentration Depend on the Transpiration Rate

**FIGURE 9:**
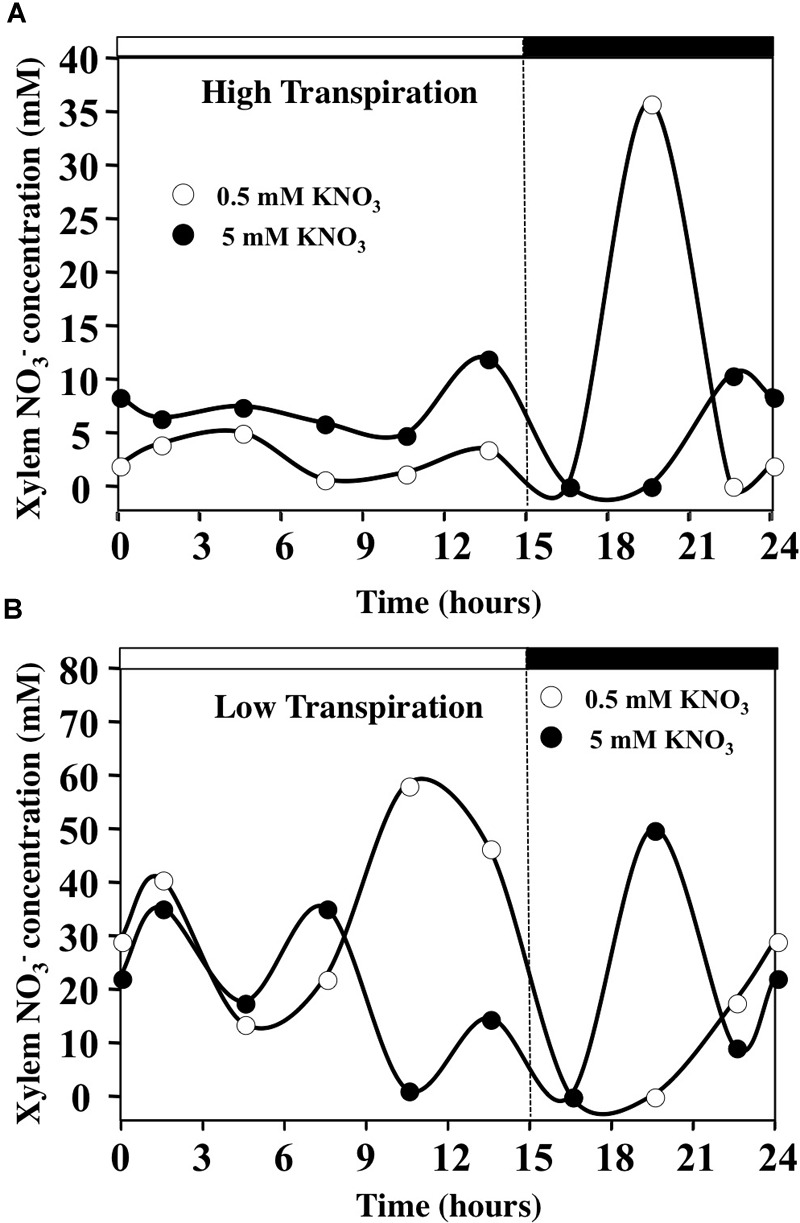
Diurnal changes in the xylem concentration of *B. napus* plants fed by 0.5 and 5 mM external nitrate concentrations and submitted to high **(A)** and low **(B)** transpiration rates. The estimates of xylem concentrations are calculated from the average values of ^15^N uptake rate and water translocation rate to the shoots.

To assess changes in xylem nitrate concentration during the day–night cycle, we estimated these concentrations from the daily variations in water uptake and ^15^NO_3_^-^ translocation rates, assuming the absence of nitrate assimilation by the roots (Figure 9 and Supplementary Figure S8). The daily patterns of nitrate concentrations in the xylem sap estimated in HT conditions were in agreement with previous data obtained in *Ricinus communis* plants fed with 0.5–12 mM of nitrate (Herdel et al., 2001; Peuke et al., 2001; Peuke, 2010). Under HT conditions for both nitrate treatments, nitrate concentration in the xylem was stable during daytime but was increased overnight with the decrease in transpiration rate and increase in ^15^N translocation to the shoot (Figure 9A and Supplementary Figure S8). However, during daytime the nitrate concentrations in xylem sap were higher in 5 mM KNO_3_ (about 5 mM) than in 0.5 mM KNO_3_ treatment (about 2.5 mM). During the night, estimates of nitrate concentrations in xylem sap were highly increased in 0.5 mM nitrate treatment with the drop in water flow, reaching a maximum value of 30 mM (Figure 9A and Supplementary Figure S8). Under LT conditions, difference between nitrate treatments was more contrasted (Figure 9B). Nitrate concentrations in xylem were very high during the day, reaching values between 20 and 35 mM, independent of external nitrate concentrations used (Figure 9B). However, in 5 mM nitrate treatment the xylem concentration decreased to 10 mM at the end of the day with the drop in ^15^N uptake (Figure 9B and Supplementary Figure S8) whereas in 0.5 mM nitrate treatment, the xylem concentration increased to 50 mM due to the increase in ^15^N uptake and the drop in water uptake (Supplementary Figure S8). When the illumination stopped, after a rapid decline in xylem nitrate concentrations for both treatments, nitrate concentrations rose again to reach values around 10–15 mM at the end of the night with the increase of ^15^N uptake and slight resumption of transpiration (Figure 9B and Supplementary Figure S8).

### Under Low Transpiration Rate and Low Nitrate Availability, the Contents of Cl^-^ and SO_4_^2-^ Anions Compensate for Diurnal Changes in Nitrate Contents of the Shoots and Roots

**FIGURE 10:**
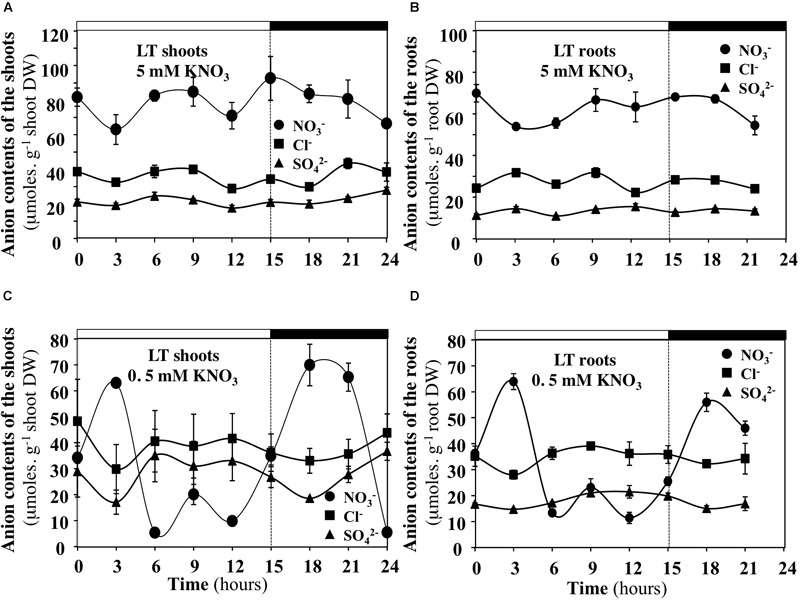
Diurnal changes in the anion contents in the shoots and roots of *B. napus* plants fed with 5 **(A,B)** and 0.5 mM **(C,D)** external nitrate concentrations at low transpiration rate. Values are the averages (±SE) of three plants (*N* = 3).

**FIGURE 11:**
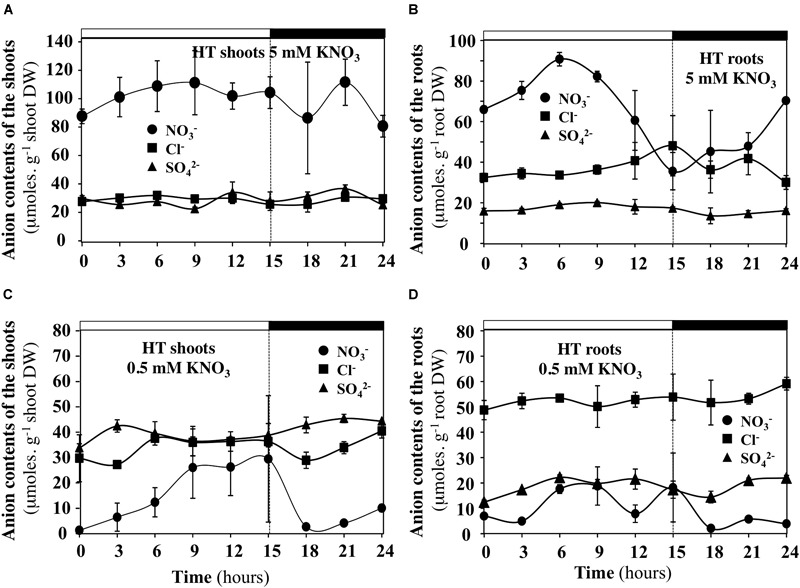
Diurnal changes in the anion contents in the shoots and roots of *B. napus* plants fed with 5 **(A,B)** and 0.5 mM **(C,D)** external nitrate concentrations at high transpiration rate. Values are the averages (±SE) of three plants (*N* = 3).

**FIGURE 12:**
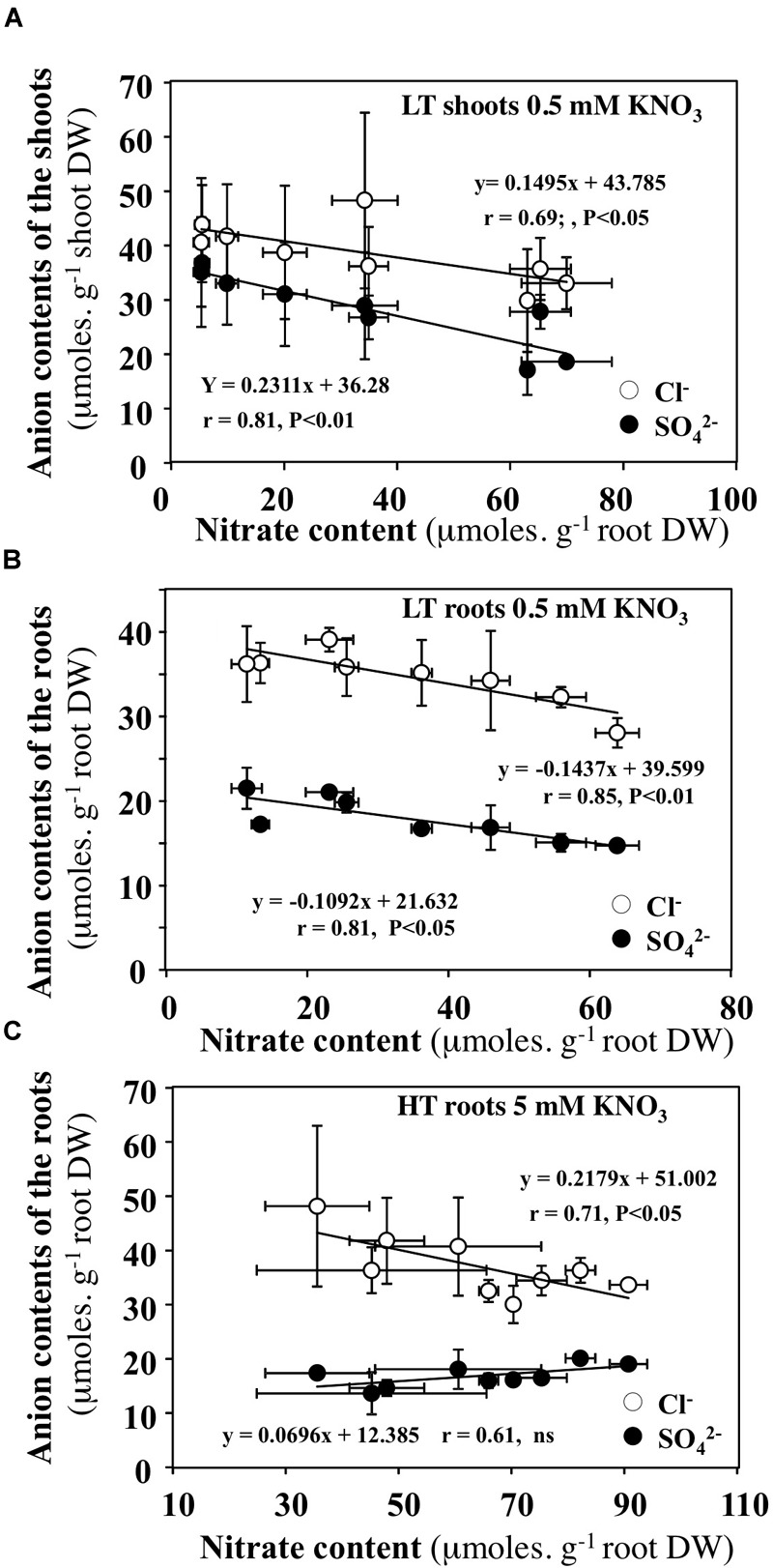
Relationships between diurnal variations in nitrate contents in the shoots and roots with chloride or sulfate contents. **(A,B)** *B. napus* plants were fed with 0.5 mM external nitrate concentrations under low transpiration rate. **(C)** *B. napus* plants were fed with 5 mM external nitrate concentrations under high transpiration rate.

In order to test whether diurnal changes in nitrate contents of the shoots and roots induced by treatments could affect content of other anions, the profiles of phosphate, malate, sulfate and chloride anions contents were measured during the diurnal course. Only profiles of chloride and sulfate contents that showed significant differences are presented with those of nitrate (Figures 10, 11). During the diurnal course, the highest amplitude changes in nitrate content in the shoots and roots were observed for plants treated with 0.5 mM nitrate under LT conditions (Figures 10C,D) and with 5 mM nitrate under HT conditions in the roots (Figure 11B). Except for these both treatments, profiles in the Cl^-^ and SO_4_^2-^ contents were not affected during the diurnal course (Figures 10, 11). In 0.5 mM nitrate under LT conditions, the shoots and roots diurnal profiles in Cl^-^ and SO_4_^2-^ contents showed strictly opposite responses to nitrate profiles (Figures 10C,D). Accordingly, negative linear relationships between diurnal changes in nitrate and Cl^-^ and SO_4_^2-^ contents were highly significant (Figures 12A,B). In contrast, in the roots treated by 5 mM nitrate under HT condition, a negative relationship was found between changes in nitrate and Cl^-^ contents whereas no significant relationship was found between changes in nitrate and SO_4_^2-^ contents (Figure 12C).

## Discussion

The main objective of this study was to highlight the rapid and fine regulation that is involved in interactions of water and nitrate fluxes during a day–night cycle in response to changes in nitrate availability and transpiration rate. This question was investigated in intact *B. napus* plants by combining the non-destructive gravimetric device developed by Van Ieperen and Madery (1994) with ^15^NO_3_^-^ labeling.

### Non-invasive Gravimetric Device Is a Powerful Tool for Analyzing Water and ^15^NO_3_^-^ Fluxes and Gain in Fresh Biomass During a Diurnal Cycle

The discontinuous recording of plants treated by different combinations of external nitrate concentrations and transpiration rates demonstrated that the non-invasive gravimetric method developed by Van Ieperen and Madery (1994) is a powerful tool to analyze the diurnal dynamic of water fluxes and gain in fresh biomass. When this method is coupled with ^15^NO_3_^-^ labeling, it allows simultaneous analysis of nitrate and water fluxes. Here we first demonstrated the capability of the device to register with high temporal resolution water and ^15^NO_3_^-^ fluxes in plants submitted to different environmental conditions. However, the main defect of the device is that the storage fluxes for growth and capacitance cannot be dissociated because of the water balance equation used (see Eq. 1, Boyer, 1985; Van Ieperen and Madery, 1994). The same limitation is also encountered with the non-invasive microwaves resonator device developed by Menzel et al. (2009) to monitor on a single plant the daily growth pattern and fresh biomass increase over several days. The limitation increases when discontinuous records are used on several plants treated simultaneously as in our study due to the inevitable shoots and roots biomass heterogeneity of the plants initially selected for the measurements. It is then difficult to combine the gain of fresh biomass with the gain of dry biomass, which remains the unique and true marker for growth. This defect can be avoided by using continuous records on several individual plants analyzed sequentially or placed in parallel devices and over several day–night cycles. Likewise, the selection of younger and more homogeneous plants should optimize the accuracy of the analyses.

### High External Nitrate Concentrations Result in Both a Significant Increase in Root Water Uptake and an Increase in Nitrate Mass Flow

In intact plants, an increase in nitrate availability from 0.5 to 5 mM nitrate induced an increase in daily water uptake (mg H_2_O root cm^-1^ 24 h^-1^) by 18,2% in HT and 52,7% in LT condition, respectively. The results confirmed previous data obtained with intact plants placed in split root systems showing that nitrate fed roots increased their daily capacities for water uptake (Guo S. et al., 2002; Li and Shao, 2003; Guo et al., 2007; Gorska et al., 2008a). The nitrate effect on root hydraulic conductance has been initially demonstrated by measurements on excised roots submitted to either free exudation (Radin and Boyer, 1982; Bigot and Boucaud, 1996; Carvajal et al., 1996; Hoarau et al., 1996; Lopez et al., 2003) or forced exudation by root pressurization (Gloser et al., 2007; Górska et al., 2010). Compared to KNO_3_, the root water uptake for growth and transpiration was slightly affected by KCl treatments at 0.5 and 5 mM. This result confirmed previous data on excised roots of maize plants where the KNO_3_ effect on root hydraulic conductance was 1.6 times higher than the effect of potassium provided by KCl (Hoarau et al., 1996). Likewise, the lack of a nitrogen source in plants treated with KCl for two days could also explain the observed differences in the shoot growth and/or capacitance. Indeed, Franco-Navarro et al. (2016) recently demonstrated that Cl^-^ ions when accumulated to macronutrient levels (>0.5 mM) regulate leaf tissues water balance and plants water relations.

Furthermore, the mean values in the rate of nitrate mass flow at 5 and 0.5 mM nitrate during the time course of the experiment were 18.4 ± 1.6 and 12.4 ± 1.4 μmoles NO_3_^-^ 3h^-1^ cm^-1^ root for HT and LT conditions, respectively (Supplementary Table S1). These values largely exceed the ratio of external nitrate concentrations (5 mM/0.5 mM = 10) expected in absence of regulation indicating that nitrate has a precocious and rapid effect on the root water uptake. The literature review shows that nitrate-induced changes in root water uptake can be explained by at least two non-exclusive physiological events: the recruitment of new functional xylem elements (Windt et al., 2006; Schulze-Till et al., 2009) and/or the increase in water channels activity (Clarkson et al., 2000; Lopez et al., 2003; Gorska et al., 2008b). Indeed, NMR imaging studies have shown that the changes in xylem water volume flow induced by high external nitrate concentration or the day–night transition are caused by an increase in the root conductive flow area corresponding to changes in the number of xylem vessels elements contributing to the water flow (Windt et al., 2006; Schulze-Till et al., 2009). Because a strong transpiration demand in rice and maize provokes a polar accumulation of aquaporins in the proximal end of root endodermis cells close to the cortical cell layer (Hachez et al., 2006; Sakurai-Ishikawa et al., 2011), it is likely that nitrate can also induce a similar relocation of water channels in endodermis cells where it induces an increase in water uptake and transpiration (Guo S. et al., 2002; Guo et al., 2007; Gorska et al., 2008a). As a result, these two conjugated effects lead to an increase in water translocation to the shoot and allow a fine and rapid adjustment of the water demand and water flow to nitrate availability.

### In HT Conditions, Nitrate Translocation to the Shoots Mainly Depends on Root Nitrate Uptake Rate

The analyses of kinetic variables indicate that under HT conditions at 0.5 and 5 mM nitrate treatment, nitrate uptake rate is one of the most critical point for ^15^N translocation to the shoots. Indeed, the mean ratio of ^15^N amounts was equal to 2.7 (Figure 8), a value that is very closed to 1.7–2.5 values estimated by the ratio in NO_3_^-^ influx rate of nitrate transport systems ([LATS + HATS]/HATS) from isotherms at 0.5 and 5 mM nitrate in *B. napus* and *Arabidopsis* plants (Filleur et al., 2001; Faure-Rabasse et al., 2002). In parallel, during the light phase, the nitrate concentrations in the shoots (Figures 11A,C) and the estimated nitrate concentrations in xylem sap (Figure 9A) remained stable. Furthermore, under steady-state conditions the cytosolic pools (cytoplasm and vacuole) size can also buffer the strongest variations in nitrate uptake rate (Britto and Kronzucker, 2001, 2003). This is exemplified in studies where alteration of nitrate sequestration in vacuole, by specific inhibitors, up regulates the expression of At-NRT1.5 nitrate transporter gene involved in xylem loading (Lin et al., 2008; Chen et al., 2012; Zhang et al., 2014; Han et al., 2016). Taken together, these results imply that in our conditions, translocation of nitrates from root to shoot depends not only on nitrate loading into the xylem, but also nitrate uptake rates resulting from HATS and LATS activities in the root.

Likewise, in our experiment, nitrate unloading in 0.5 and 5 mM nitrate treatment under HT condition is not a limiting step. This is consistent with the stability of shoot nitrate concentrations (Figures 11A,C) and the mean ratio of ^15^N amounts that were translocated to the shoots between 5 and 0.5 mM treatment (Figure 8). Again, the value equal to 2.7 indicated that the xylem unloading appears to depend mainly on the ratio of nitrate uptake rates at root level. In line with these results, several papers have demonstrated that xylem unloading does not constitute a limiting step since the rate of nitrate assimilation in leaves during the first part of the light period greatly exceeds the rate of nitrate uptake and transfer to the leaves (Shaner and Boyer, 1976; Matt et al., 2001). In addition, it has been reported that nitrate transporters At-NPF7.2 (NRT1.8) and Os-NPF2.2 with a low nitrate affinity and high transport capacity are specifically expressed in the xylem parenchyma and are mainly involved in the xylem unloading (Li et al., 2010, 2015). Both genes are up regulated by nitrate and the knockout mutants showed an increase of nitrate in xylem sap. In this context, the large differences in shoot nitrate concentrations measured at 0.5 and 5 mM nitrate (Figures 11A,C) are likely to be buffered by high capacities for nitrate storage and assimilation of the shoots compared to the roots (Chiu et al., 2004; Nikolic et al., 2012; Leblanc et al., 2013; Le Ny et al., 2013).

### In LT Conditions, Nitrate Translocation to the Shoots Depends on Both Root Nitrate Uptake Rate and Nitrate Loading Into the Xylem

Under LT condition, similar comparisons lead to different conclusions. First of all, the mean ratio of ^15^N amounts in the plant and shoots is around 1–1.2 indicating that LATS transport system is down regulated in 5 mM treatment whereas HATS transport system is up-regulated (Figure 8 and Supplementary Table S2). Secondly, significant variations of nitrate concentrations in the xylem sap were observed between 0.5 and 5 mM treatments at the end of light period between 7.5 and 15 h (Figure 9B). Thirdly, during 5 mM nitrate treatment, the nitrate concentrations in shoot tissues remained high and stable (Figures 10A,B) whereas during 0.5 mM treatment, nitrate concentrations were highly fluctuating (Figures 10C,D). In particular, during nitrate treatment at 0.5 mM when nitrate concentrations collapsed in the shoots and roots between 6 and 15 h (Figures 10C,D), there was a concomitant increase in xylem nitrate concentrations (Figure 9B). Taken together these results indicate that regulation of the N translocation may occur either at the loading step of nitrate into the xylem and/or during the radial transport of nitrate across the root. The strong expression of At-NRT1.1 and At-NRT2.1 genes in root epidermis and endodermis supports this last assumption. This hypothesis is also consistent with a greater expression of At-NRT1.1 in endodermis (Guo F.Q. et al., 2002; Nazoa et al., 2003) and the major role played by At-NRT2.1 in roots hydraulic conductivity (Li et al., 2016).

With respect to nitrate unloading, during 0.5 mM nitrate treatment between 6 and 15 h, the parallel collapse of nitrate concentrations observed in the shoots and roots was offset by an increase in SO_4_^2-^ and Cl^-^ concentrations. This anion exchange probably resulted in a better use of nitrate to meet the high N demand required for shoot growth (Figures 10C,D). Again, this suggests that xylem unloading is not a limiting step under LT conditions.

In summary, the nitrate xylem loading is potentially a limiting step only at 0.5 mM nitrate treatment under LT conditions whereas the unloading of nitrate is never a limiting step whatever the conditions used. Although these observations contrast with a previous study in *R. communis* (Herdel et al., 2001), they confirm the conclusions obtained by Delhon et al. (1995b) in both excised and intact plants of soybean. This is also consistent with the positive feedback regulation exerted by nitrate on the gating of X-QUAC channels during xylem loading, involvement of At-NRT2.1 transporter in the root hydraulic conductivity and induction by nitrate of a greater number of xylem vessels involved in water flow (Köhler and Raschke, 2000; Köhler et al., 2002; Schulze-Till et al., 2009; Li et al., 2016).

### The Combination of Transpiration Rate and Nitrate Availability Modulates Diurnal Profiles in Water Accumulation Rates in the Shoots

The daily gain of fresh weight showed an increase in the rate of the tissues capacitance mainly from the end of the day and during night. Similar pattern was observed in tomato plants when using the same gravimetric device (Van Ieperen and Madery, 1994) and a microwave resonator (Menzel et al., 2009). This behavior was characteristic of a daily leaf growth pattern of dicotyledonous type 1 plants such as *Arabidopsis* (Ruts et al., 2012). However, the combination of macronutrient concentrations and RH conditions during diurnal course indicated that the intensity and duration of shoot water accumulation rate were modulated by changes in external nitrate concentrations (Supplementary Figure S5). The increase in nitrate concentration from 0.5 to 5 mM induced a significant increase in water accumulation and provoked a phase shift in the shoot water accumulation rate toward the onset of the light period (from 13.5 to 9 h). In addition, the transition between HT and LT conditions amplifies this behavior and led to a significant increase in the maximum water accumulation rate during the light phase (e.g., 135 in HT to 257 mg. h^-1^. plant^-1^ in LT at 5 mM). These results are very intriguing because they seem indicate that the circadian clock of plants is under control of nitrate availability or some N-derived metabolites. If they are consistent with recent results in *Arabidopsis*, which showed that the phase of CAA1 gene of the master clock (Circadian Clock Associated 1) is advanced or delayed by different N metabolites such as glutamate or glutamine (Gutiérrez et al., 2008), they deserve more investigations. Thus, the effect of nitrate on the biological clock and CAA1 expression could be easily verified with the gravimetric device by using pharmacological treatments such as MSX (methionine sulfoximine) to inhibit the activity of glutamine synthase (GS) or mutants of the glutamate receptors, glutamine synthase, aquaporins and nitrate carriers.

### Compensations by Chloride Ions for Large Changes of Nitrate Concentrations in Bulk Tissue Extracts Are in Agreement With Non-selectivity of Some Anion Channels for Cl^-^ and NO_3_^-^

Chloride and nitrate are monovalent anions with a similar ionic radius that can be transported in short and long distance by the same transporters such as CLCs (ChLoride Channels) and SLAC/SLAH (SLow-type Anion Channels/SLow-type Anion channels Homolog) channels (for reviews see Li et al., 2017; Wege et al., 2017). The well-known antagonistic effects of Cl^-^ on the root nitrate uptake, osmotic regulation and charge balance functions at vacuolar level have been recently revised (Glass and Siddiqi, 1985; Flowers, 1988; Cubero-Font et al., 2016). As suggested by Flowers (1988), the high accumulation of Cl^-^ in the vacuole would prevent the diversion of important nutrients such as nitrate for the general metabolism. This hypothesis was recently supported by the positive effect of chloride treatment on shoot growth in tobacco plants. This result was not observed with the anionic macronutrients such as nitrate, sulfate and phosphate despite the same cationic balance in mediums composition (Franco-Navarro et al., 2016). Accordingly, plants would preferentially use Cl^-^ for osmotic regulation when Cl^-^ availability is in the macronutrient range (>0.5 mM) thus promoting a more efficient use of nitrogen. In contrast, NO_3_^-^ would be used as an osmolyte or charge-balancing molecule when Cl^-^ availability is in the micronutrient range (<0.1 mM; Franco-Navarro et al., 2016). Our data clearly support the NO_3_^-^ diversion hypothesis. Furthermore, they demonstrate that there exists during diurnal course, a fine and rapid interchange in Cl^-^ and NO_3_^-^ ions allowing osmotic regulation and changes in tissues capacitance and growth in order to buffer changes in nutrient availability and transpiration rate. This was particularly evident for the treatment with 0.5 mM nitrate under LT condition between 6 and 15h during the light period where the low levels of nitrate contents in the shoots and high N demand for metabolism were both balanced by high nitrate concentrations in xylem sap (reaching up to 60 mM) and an increase in chloride contents in roots and shoots. Taken together, all these results are in agreement with the non-selectivity of Cl^-^/NO_3_^-^ transporters such as CLC and SLAC/SLAH channels (Geiger et al., 2009, 2011; Zifarelli and Pusch, 2009; Maierhofer et al., 2014; Cubero-Font et al., 2016) involved in short and long distance transport of Cl^-^ and NO_3_^-^ and their role in plant growth and rapid adjustment of water relations (Franco-Navarro et al., 2016; Wege et al., 2017). Although it is clear from the experiment that there is a fine and rapid exchange of Cl^-^ and NO_3_^-^ during diurnal course, however, in nature this is unlikely as chloride is not so abundant in soil. Therefore, the role of SO_4_^2-^ will need to be re-evaluated in the future, as there is also a strong correlation between SO_4_^2-^ and NO_3_^-^ in LT condition.

## Conclusion

The non-invasive gravimetric device used in this study coupled with ^15^NO_3_^-^ labeling is a powerful tool for diurnal kinetic analysis of water and nitrate fluxes for plant growth and capacitance by using high resolution time-lapse weighing of individual seedling. The use of mutants of water channels, nitrate, chloride and sulfate transporters combined with different root pharmacological treatments will help to identify the candidate genes responsible for the fine and rapid diurnal regulations of nitrate and water fluxes involved in nitrogen and water use efficiency (NUE and WUE).

## Author Contributions

ELD, M-LD, and CO designed the research. ELD, CO, GD, and M-PB performed the research. PB performed the LiCor 6400 analyses. ELD, CO, and GD analyzed the data. ELD and M-LD wrote the paper.

## Conflict of Interest Statement

The authors declare that the research was conducted in the absence of any commercial or financial relationships that could be construed as a potential conflict of interest.
