# Supplementary figures and images for: Changes in 15NO3- Availability and Transpiration Rate Are Associated With a Rapid Diurnal Adjustment of Anion Contents as Well as 15N and Water Fluxes Between the Roots and Shoots
Source: Front Plant Sci. 2018 Dec 3;9:1751. doi: 10.3389/fpls.2018.01751 (PMC6287045; doi:10.3389/fpls.2018.01751)

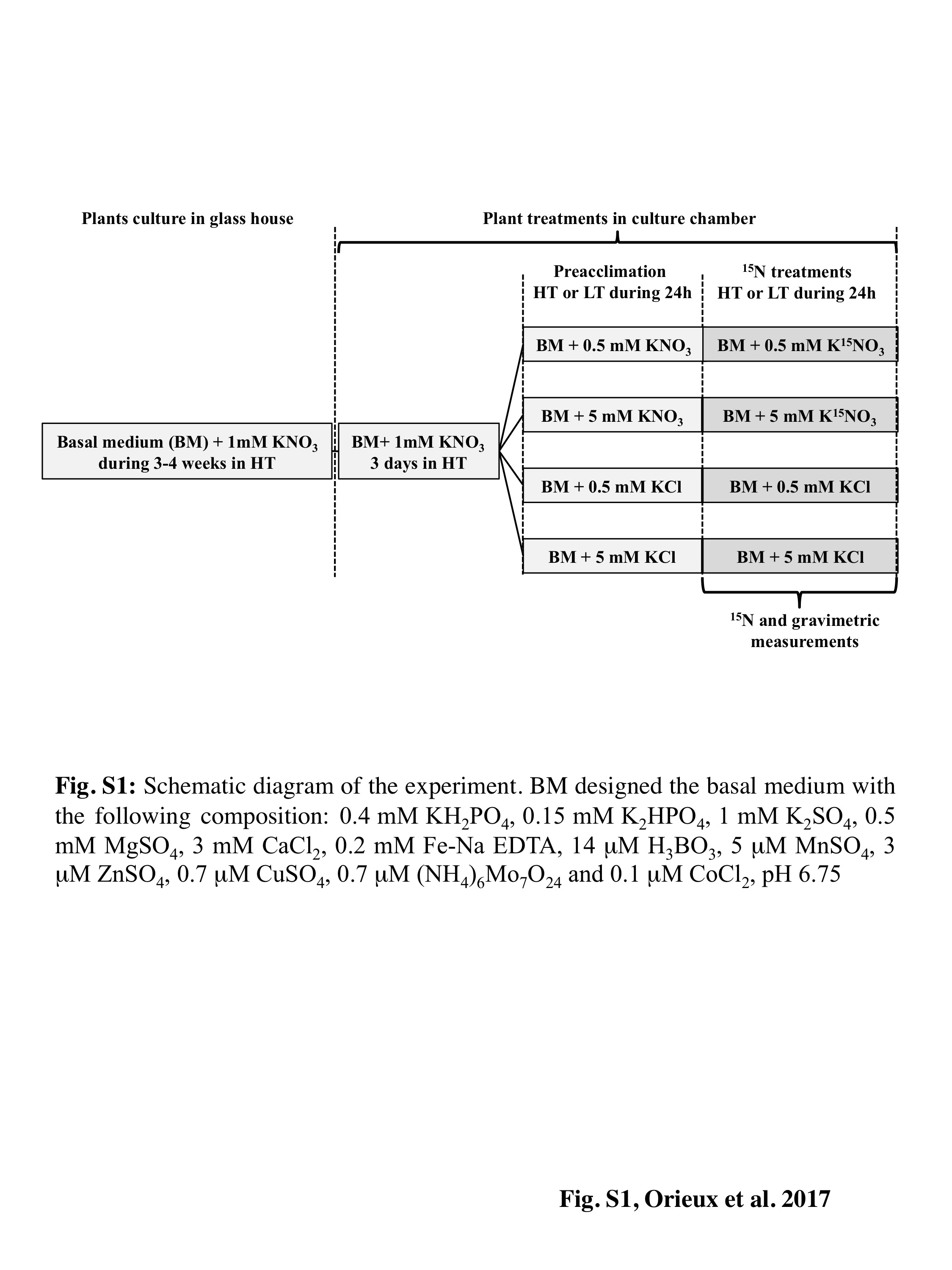

Supplement: Supplementary file 1 [file Image_1.JPEG]

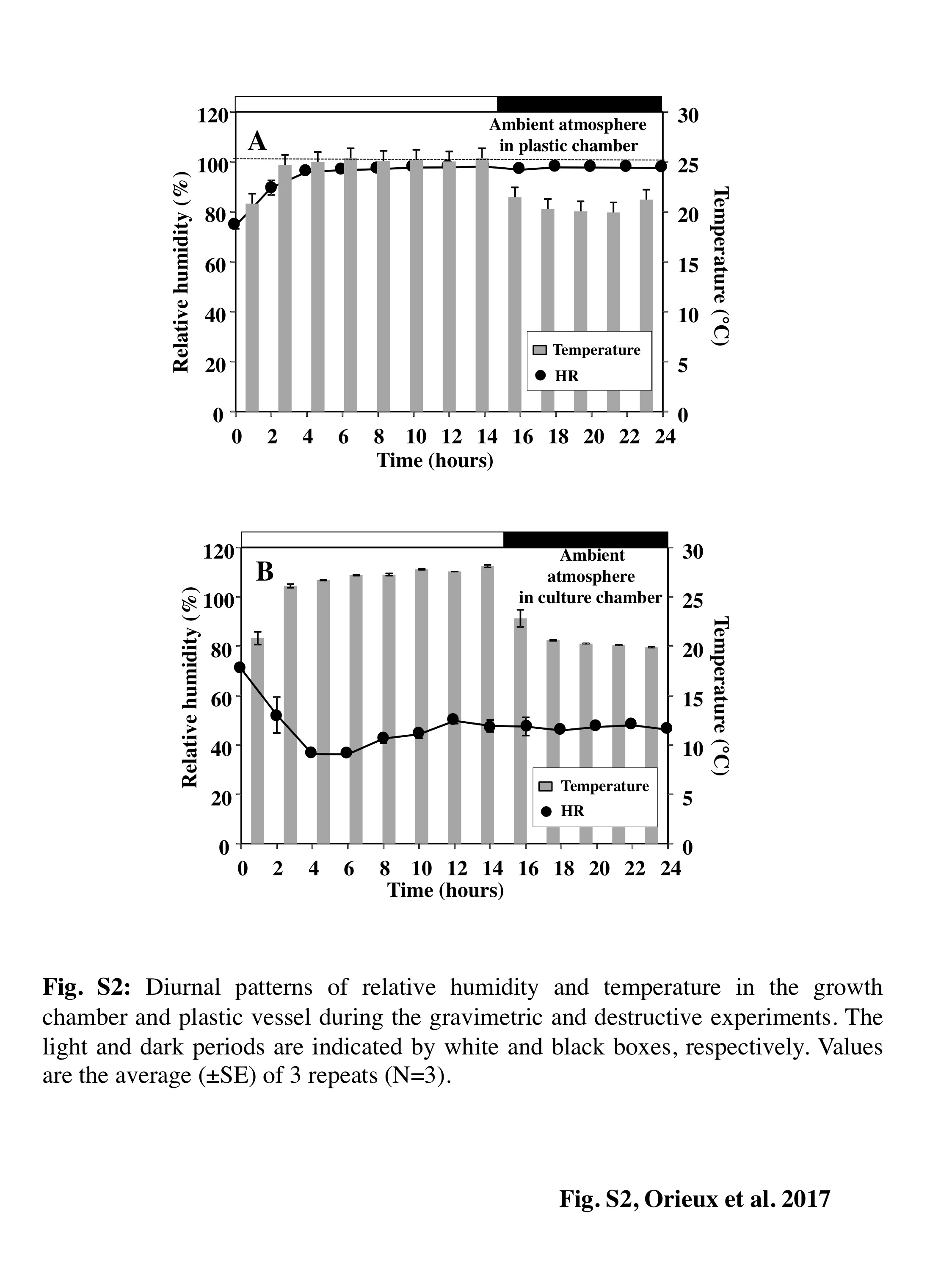

Supplement: Supplementary file 2 [file Image_2.JPEG]

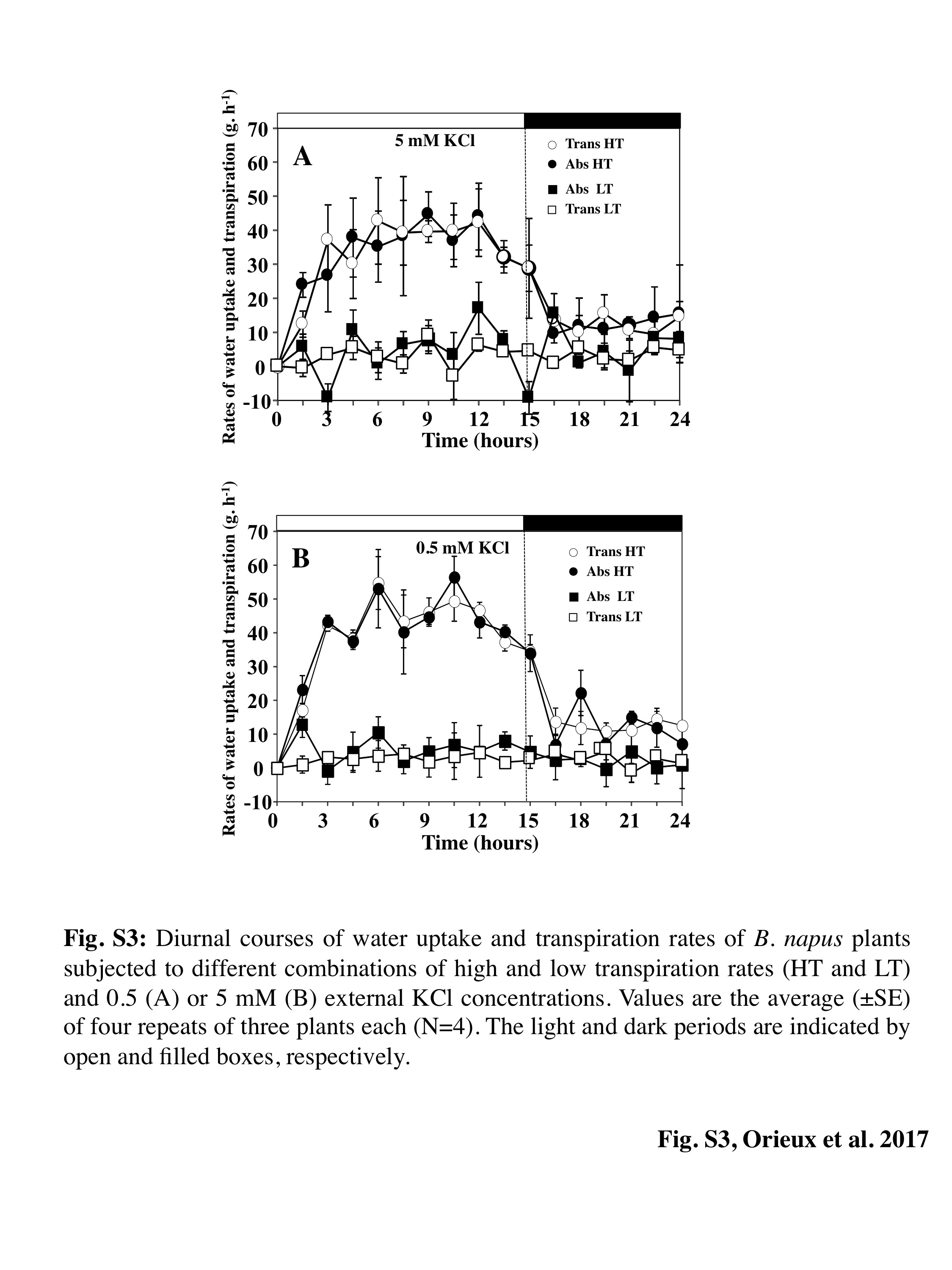

Supplement: Supplementary file 3 [file Image_3.JPEG]

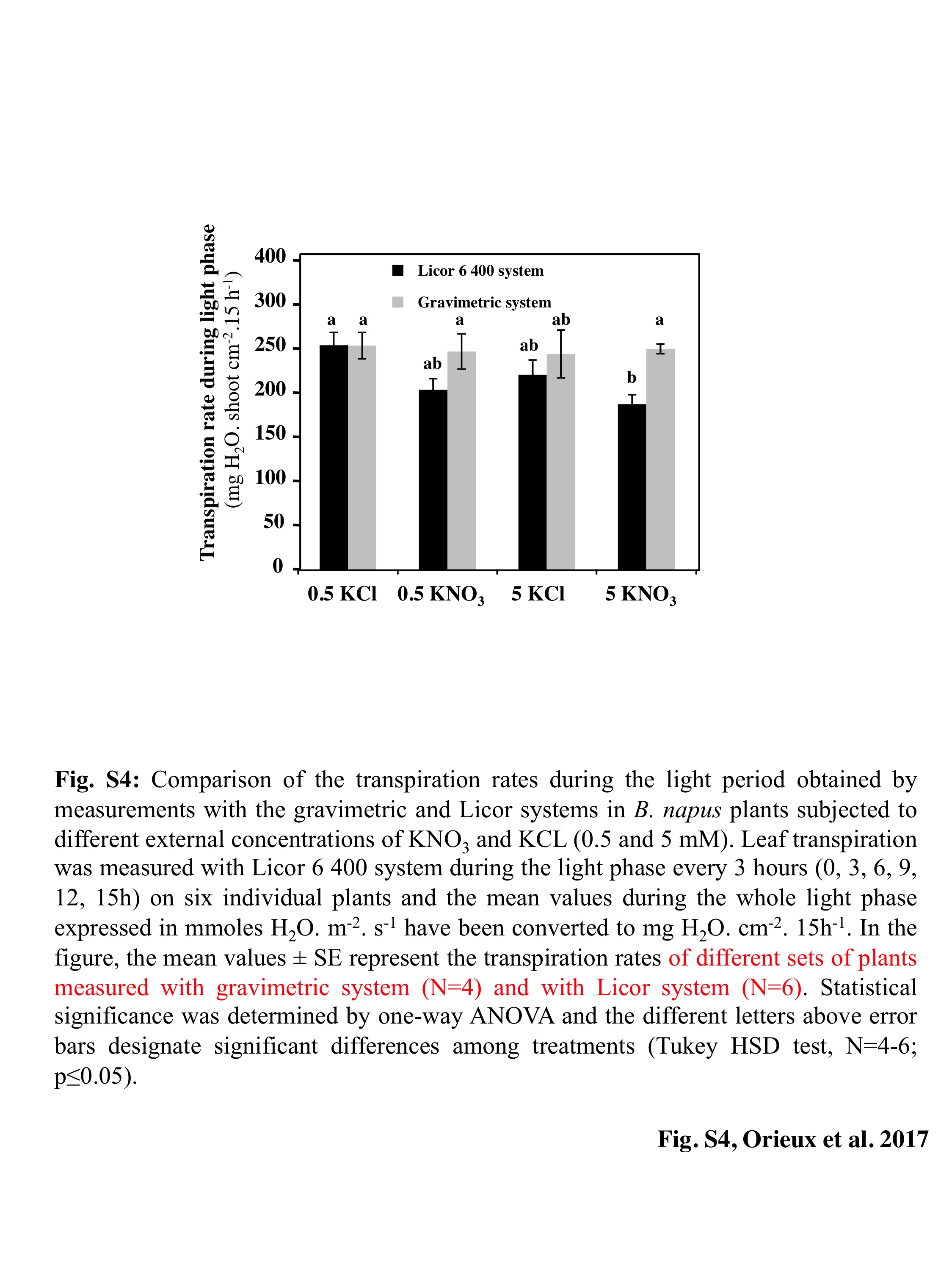

Supplement: Supplementary file 4 [file Image_4.jpg]

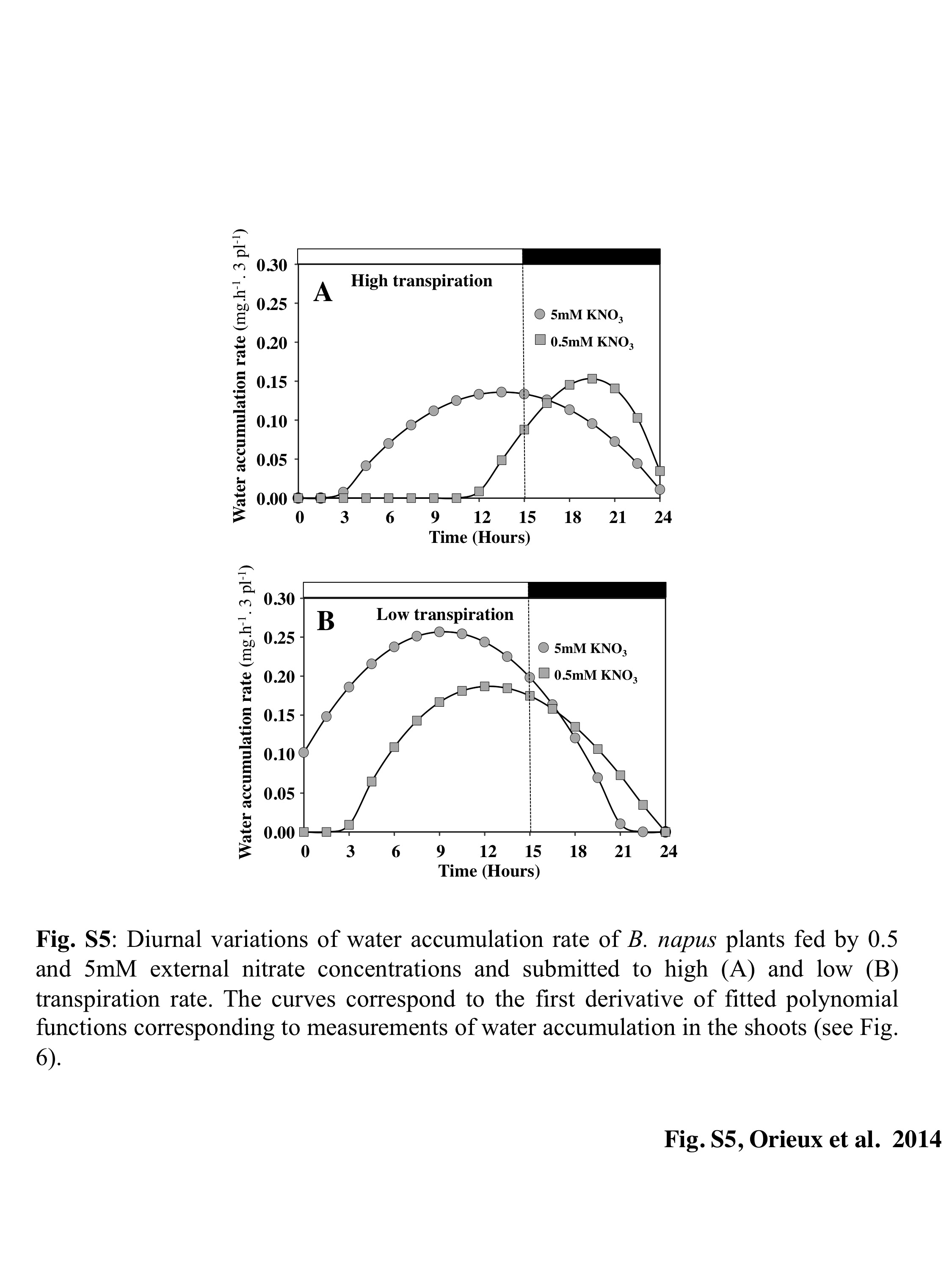

Supplement: Supplementary file 5 [file Image_5.JPEG]

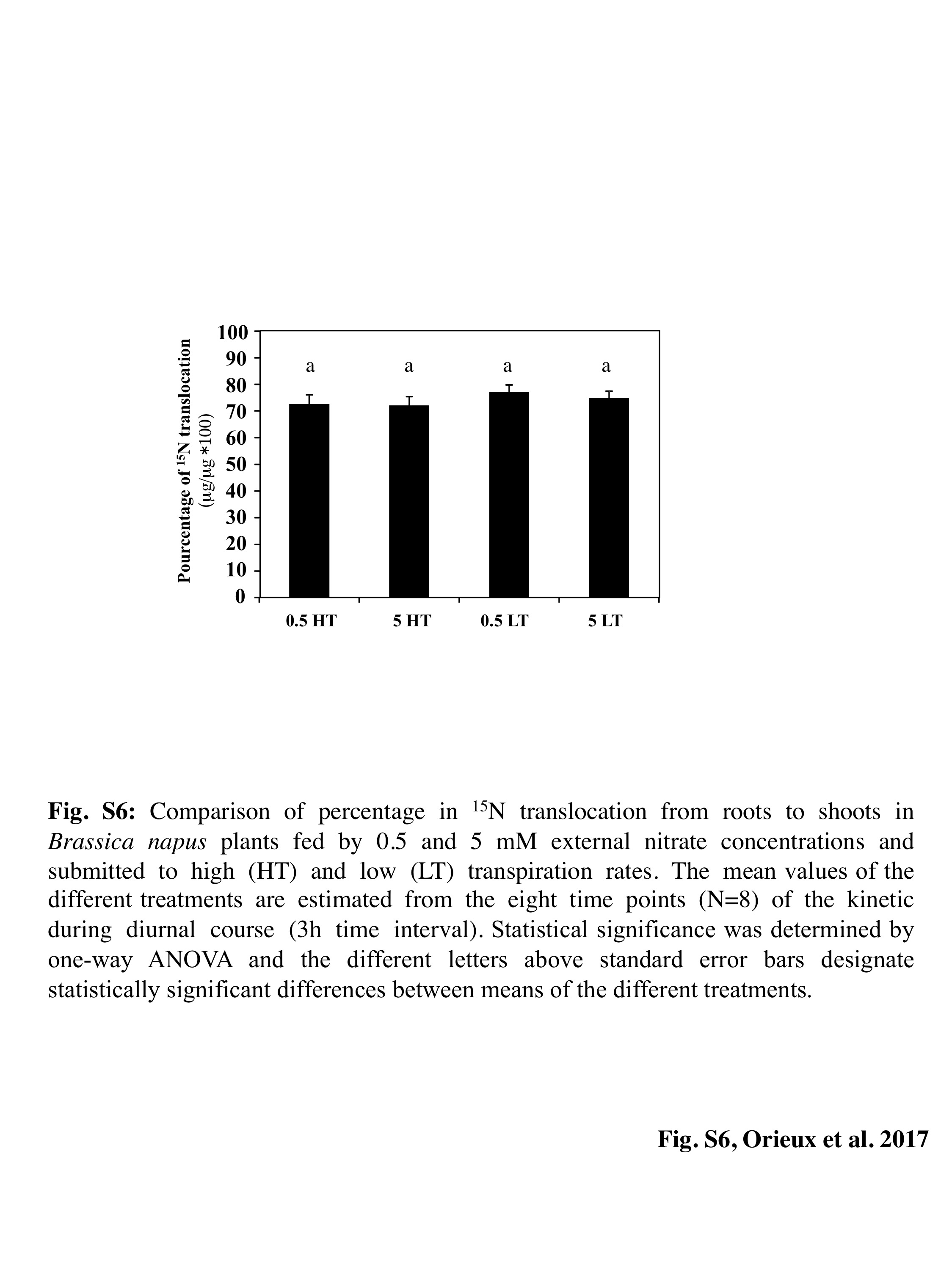

Supplement: Supplementary file 6 [file Image_6.JPEG]

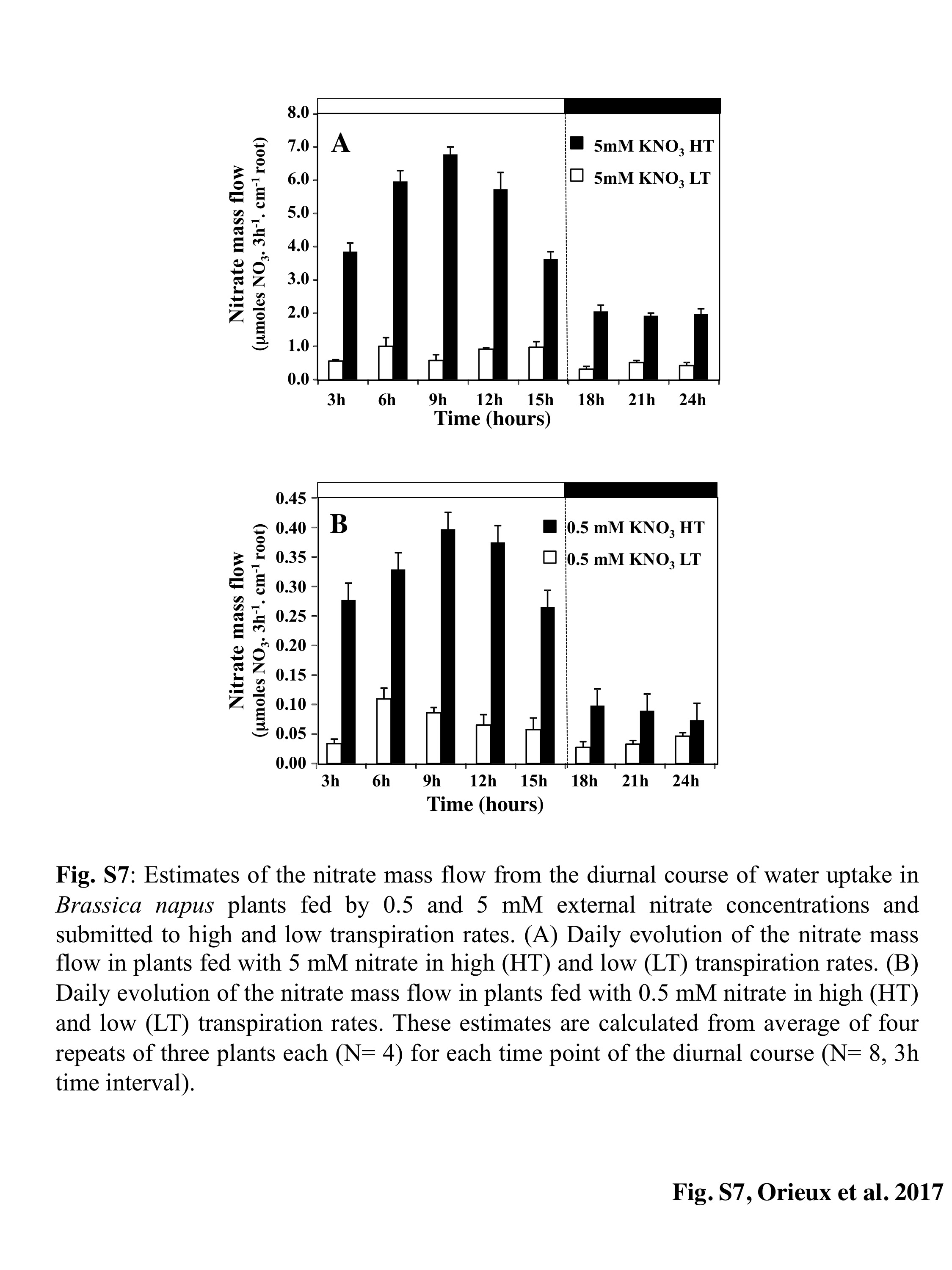

Supplement: Supplementary file 7 [file Image_7.JPEG]

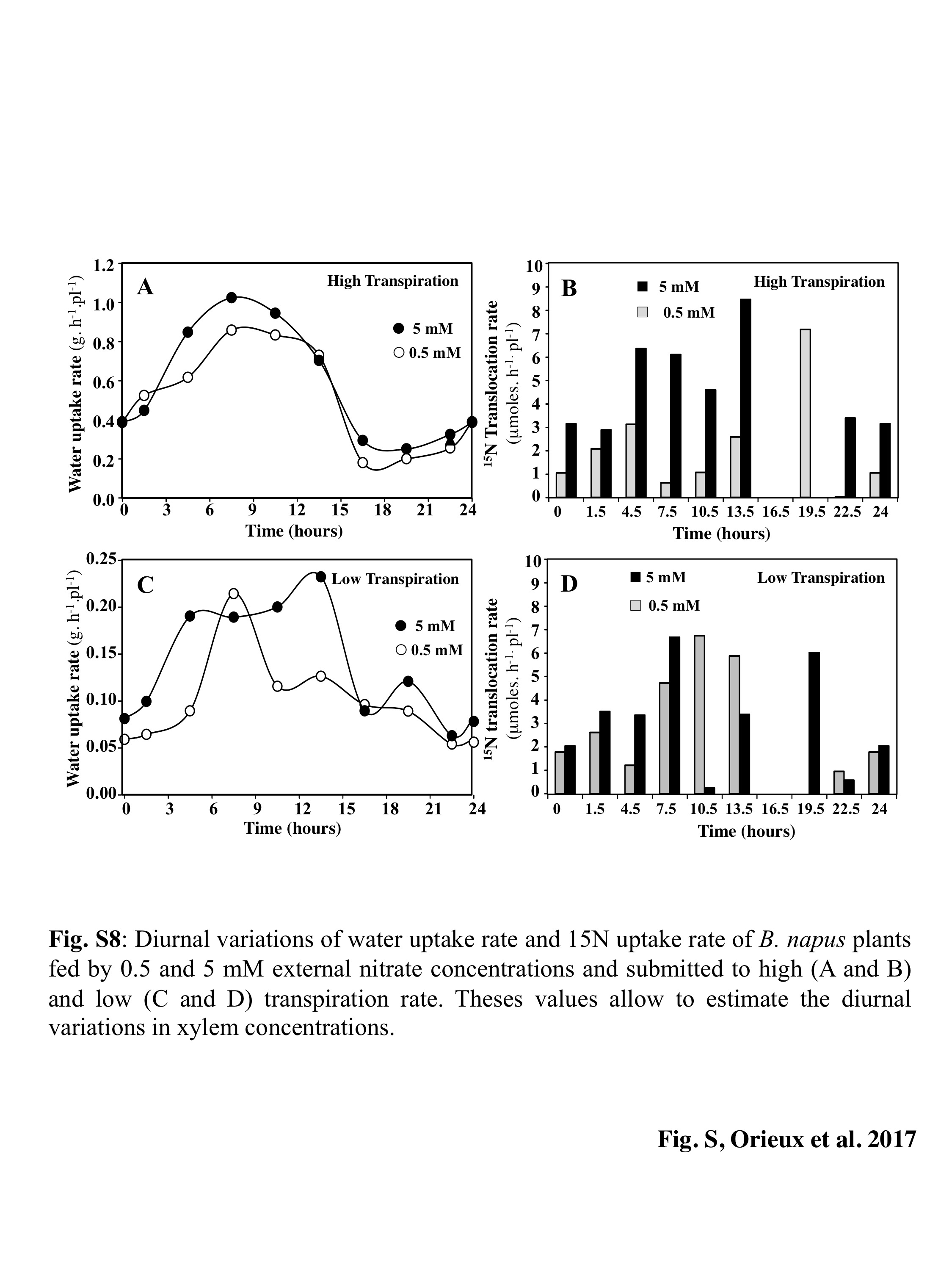

Supplement: Supplementary file 8 [file Image_8.JPEG]
