# Supplementary material for: Changes in 15NO3- Availability and Transpiration Rate Are Associated With a Rapid Diurnal Adjustment of Anion Contents as Well as 15N and Water Fluxes Between the Roots and Shoots
Source: Front Plant Sci. 2018 Dec 3;9:1751. doi: 10.3389/fpls.2018.01751 (PMC6287045; doi:10.3389/fpls.2018.01751)
Supplement: Supplementary file 9 [file Table_1.DOCX]

**Table S1**: Mean ratio of the nitrate mass flow of *Brassica napus* plants fed by 0.5 and 5 mM external nitrate concentrations and submitted to high (HT) and low (LT) transpiration rates. Values are the average (±SE) of four repeats of three plants each for each time point (N=4) of the daily kinetic.

|  | **High transpiration rate**  Nitrate mass flow in μmoles NO_3_^-^. 3h^-1^. cm^-1^ root | | | **Low transpiration rate**  Nitrate mass flow in μmoles NO_3_^-^. 3h^-1^. cm^-1^ root | | |
| --- | --- | --- | --- | --- | --- | --- |
| **Time** | **5mM KNO_3_** | **0.5mM KNO_3_** | **Ratio**  **5 / 0.5** | **5mM KNO_3_** | **0.5mM KNO_3_** | **Ratio**  **5 / 0.5** |
| **0-3h** | 3.84 ± 0.25 | 0.28 ± 0.02 | 13.9 | 0.56 ± 0.42 | 0.03 ± 0.008 | 16.3 |
| **3-6h** | 5.97 ± 0.32 | 0.33 ± 0.03 | 18.2 | 1.00 ± 0.26 | 0.10 ± 0.018 | 9.1 |
| **6-9h** | 6.78 ± 0.22 | 0.40 ± 0.04 | 17.1 | 0.57 ± 0.18 | 0.08 ± 0.009 | 6.6 |
| **9-12h** | 5.72 ± 0.51 | 0.37 ± 0.04 | 15.3 | 0.92 ± 0.03 | 0.06 ± 0.017 | 14.1 |
| **12-15h** | 3.63 ± 0.22 | 0.26 ± 0.02 | 13.7 | 0.97 ± 0.17 | 0.06 ± 0.020 | 16.9 |
| **15-18h** | 2.06 ± 0.19 | 0.09 ± 0.01 | 21.0 | 0.31 ± 0.08 | 0.03 ± 0.010 | 11.4 |
| **18-21h** | 1.92 ± 0.08 | 0.09 ± 0.02 | 21.5 | 0.51 ± 0.06 | 0.03 ± 0.006 | 15.4 |
| **21-24h** | 1.97 ± 0. 17 | 0.07 ± 0.02 | 26.7 | 0.42 ± 0.1 | 0.04 ± 0.006 | 9.1 |
| **Mean ± SE** |  |  | **18.4 ± 1.57** |  |  | **12.37 ± 1.36** |
