# Supplementary material for: Changes in 15NO3- Availability and Transpiration Rate Are Associated With a Rapid Diurnal Adjustment of Anion Contents as Well as 15N and Water Fluxes Between the Roots and Shoots
Source: Front Plant Sci. 2018 Dec 3;9:1751. doi: 10.3389/fpls.2018.01751 (PMC6287045; doi:10.3389/fpls.2018.01751)
Supplement: Supplementary file 10 [file Table_2.DOCX]

**Table S2**: ^15^N accumulation in roots and shoots of *Brassica napus* plants fed by 0.5 and 5 mM external nitrate concentrations and submitted to high (A) and low (B) transpiration rates. Values are the average (±SE) of 3-6 plants for each time point of the diurnal ^15^N accumulation profiles.

| **B** | **0.5 mM KNO_3_ + Low transpiration rate**  **^15^**N accumulation in μg. organ**^-1^** | | | **5 mM KNO_3_ + Low transpiration rate**  **^15^**N accumulation in μg. organ**^-1^** | | |
| --- | --- | --- | --- | --- | --- | --- |
| **Time** | **Roots** | **Shoots** | **Plant** | **Roots** | **Shoots** | **Plant** |
| **0-3h** | 88.6 ± 14.5 | 117.6 ± 15.9 | 206.2 ± 25.9 | 92.2 ± 15.6 | 157.8 ± 25 | 250.1 ± 39.6 |
| **3-6h** | 50.7 ± 5.7 | 172.3 ± 26.5 | 223 ± 26.6 | 98.6 ± 13.1 | 309.1 ± 40.5 | 407.7 ± 50.9 |
| **6-9h** | 93.5 ± 24.9 | 385.3 ± 78.5 | 478.8 ± 97.5 | 141.3 ± 19.9 | 609.7 ± 74.1 | 750.7 ± 92 |
| **9-12h** | 135.1 ± 10.2 | 688.7 ± 64.6 | 823.7 ± 69.1 | 253.3 ± 26.5 | 620.4 ± 56.1 | 873.7 ± 75.2 |
| **12-15h** | 222.5 ± 17.7 | 953.5 ± 31.5 | 1175.9 ± 35.9 | 346.7 ± 43.8 | 772.9 ± 64.1 | 1119.5 ± 78.8 |
| **15-18h** | 229.5 ± 47.9 | 903.6 ± 194.8 | 1132.8± 175.7 | 279.7 ± 37.7 | 707.9 ± 38.1 | 987.7 ± 53.3 |
| **18-21h** | 238.3 ± 62.5 | 847.7 ± 102.3 | 1085.9 ± 23 | 217.5 ± 41.3 | 979.2 ± 72.6 | 1196.8 ± 101 |
| **21-24h** | 268.9 ± 65.5 | 890.6 ± 18.2 | 1159.5 ± 62.8 | 204.7 ± 11.2 | 1005.4 ± 121.4 | 1210.1 ± 128.8 |

| **A** | **0.5 mM KNO_3_ + High transpiration rate**  **^15^**N accumulation in μg. organ**^-1^** | | | **5 mM KNO_3_ + High transpiration rate**  **^15^**N accumulation in μg. organ**^-1^** | | |
| --- | --- | --- | --- | --- | --- | --- |
| **Time** | **Roots** | **Shoots** | **Plant** | **Roots** | **Shoots** | **Plant** |
| **0-3h** | 88.5 ± 26.6 | 93.9 ± 11.5 | 182.4 ± 24.6 | 85.2 ± 021.7 | 130.1 ± 18.4 | 264.1 ± 35.3 |
| **3-6h** | 152.6 ± 19 | 234.8 ± 27.4 | 387.5 ± 40.5 | 399.3 ± 130.5 | 416.5 ± 132.8 | 640.6 ± 208 |
| **6-9h** | 106.7 ± 10.9 | 263.2 ± 36.1 | 369.9 ± 43.9 | 207.7 ± 38.9 | 691.5 ± 134.2 | 899.2 ± 165.9 |
| **9-12h** | 69.1± 20 | 311.6 ± 68.1 | 380.7 ± 62.2 | 227.3 ± 23.5 | 898.5 ± 127.5 | 1038.9 ± 178.4 |
| **12-15h** | 104.9 ± 20.8 | 428.2 ± 42 | 533.3 ± 61.2 | 411.6 ± 141.4 | 1279.6 ± 245 | 1691.2 ± 278.5 |
| **15-18h** | 97.5 ± 29.3 | 323.8 ± 33.1 | 421.3 ± 56.8 | 355.4 ± 93.3 | 1259.9 ± 183 | 1615.3 ± 126.4 |
| **18-21h** | 150.6 ± 36.7 | 647.3 ± 134.5 | 818 ± 157 | 334.6 ± 41.8 | 1071.9 ± 93.5 | 1322.8 ± 142.5 |
| **21-24h** | 139.5 ± 20.5 | 648.8 ± 76.5 | 803.2 ± 62.1 | 436.4 ± 124.4 | 1224.8 ± 251.6 | 1661.2 ± 363 |
